# MiR-142-3p targets the CXCL12/WNT/β-catenin pathway to regulate the stemness of breast cancer cells

**DOI:** 10.1038/s41598-025-34163-4

**Published:** 2025-12-29

**Authors:** Jing Wang, Qiong Wu, Zhaojun Zhang, Miaomiao Xie, Ke Wei, Wenrui Wang, Qingling Yang, Yurong Shi

**Affiliations:** 1 Anhui Provincical Key Laboratory of Tumor Evolution and Intelligent Diagnosis and Treatment, Bengbu Medical University, Bengbu, Anhui, 233000 China; 2 Department of Laboratory Medicine, the Fuyang Affiliated Hospital of Anhui Medical University, Fuyang, Anhui 236000 China; 3 Department of Biotechnology, Bengbu Medical University, Bengbu, Anhui, 233000 China; 4 Department of Biochemistry and Molecular Biology, Bengbu Medical University, Bengbu, Anhui, 233000 China

**Keywords:** Breast cancer, miR-142-3p, CXCL12, EMT, Stemness, Breast cancer, Breast cancer

## Abstract

Breast cancer is the most common type of cancer among women. It is well-established that microRNAs (miRNAs) play a critical role in cancer development by either degrading messenger RNA (mRNA) or inhibiting its translation, thereby suppressing the expression of specific genes. In this study, we found that the expression level of miR-142-3p was significantly lower in breast cancer cells and tissues than in normal breast epithelial cells and adjacent tissues. We demonstrated that miR-142-3p could inhibit the proliferation, migration, epithelial-mesenchymal transition (EMT), and stemness of MCF 7 breast cancer cells, while also promoting apoptosis. Further investigation revealed that miR-142-3p directly targets CXCL12 and regulates its expression. Silencing CXCL12 (using CXCL12 siRNA) suppressed the migration, EMT, and stemness of MCF 7 cells, and these effects were reversed by inhibition of miR-142-3p. Additionally, we observed alterations in β-catenin protein levels, suggesting that miR-142-3p may modulate the WNT/β-catenin signaling pathway through targeting CXCL12 in MCF 7 cells. Subsequent experiments indicated that miR-142-3p also plays a crucial role in overcoming paclitaxel resistance in MCF 7/PTX cells. SOX2 protein levels, which are associated with paclitaxel resistance, proliferation, migration, and EMT, were higher in MCF 7/PTX cells compared to MCF 7 cells. Overall, our findings suggest that miR-142-3p influences breast cancer progression by targeting the CXCL12/WNT/β-catenin pathway, thereby affecting cell migration, EMT, stemness, and paclitaxel resistance.

**Supplementary Information:**

The online version contains supplementary material available at 10.1038/s41598-025-34163-4.

## Introduction

Breast cancer is now the most common form of cancer among women, accounting for 31% of all new cancer diagnoses in females^1^. Despite various preventive and treatment strategies in clinical settings, challenges such as recurrence, distant metastasis, and drug resistance continue to complicate breast cancer management. The epithelial-mesenchymal transition (EMT) plays a critical role in cancer metastasis and invasion. Additionally, it is associated with the stemness and treatment resistance observed in breast cancer^2^. Current treatment modalities for breast cancer fall are unable to completely eradicate all cancerous cells, particularly breast cancer stem cells (BCSCs)^3^. The core transcriptional network of OCT4, SOX2, and NANOG is crucial for the self-renewal and pluripotency of cancer stem cells (CSCs), and studies have shown that their expression levels are higher in various cancers compared to normal tissues. Elevated expression levels are associated with advanced cancer stages, poor prognosis, and reduced survival rates^4^. Therefore, it is necessary to study regulatory factors that affect the stem cell characteristics of cancer cells.

MicroRNA (miRNA) represents a class of abundant, small, endogenous RNA molecules, ranging in lengthfrom 18 to 22 nucleotides. They function as non-coding post-transcriptional regulators of gene expression. Dysregulation of miRNA expression can affect tumor cell proliferation, apoptosis, metastasis, and drug resistance^5–7^. Recently, Zou et al. discovered that that miR-6836delivered via exosomes, can enhance ovarian cancer cells’ resistance to cisplatin by increasing cancer cell stem-like properties and inhibiting apoptosis^8^. They also observed that miR-142-3p was expressed at low levels in breast cancer cells and tissues. Although previous studies indicated that miR-142-3p could inhibit autophagy by targeting the GNB2/AKT/mTOR signaling pathway, thereby enhancing breast cancer resistance to paclitaxel^9^, the mechanisms by which miR-142-3p regulates EMT and stemness in breast cancer cells remain largely unexplored.

CXCL12 is a key chemokine in the tumor microenvironment (TME), and its high expression is associated with high risk and poor prognosis in various common cancers, including breast cancer, hepatocellular carcinoma, and colorectal cancer^10–12^. Prior studies, including our own, have shown that miRNA-7, miR-155-3p, and miR-155-5p play roles in influencing breast cancer metastasis and drug resistance by regulating the CXCL12/CXCR4 signaling pathway^13,14^. The CXCL12/CXCR4 axis is known to promote EMT through mTOR activation, while Dipeptidyl Peptidase-4 (DPP-4) can counteract EMT by cleaving CXCL12. Inhibition of DPP-4 is associated with increased metastasis in vivo^15^. Furthermore, exosomal miR-372-5p can affect of M2 macrophages polarization and increase CXCL12 secretion, thereby promoting the stemness and metastasis of colorectal cancer cells^16^. Despite these findings, the specific molecular mechanisms by which CXCL12 regulates EMT and stemness in breast cancer cells still need further elucidation.

In this study, we observed that the expression of miR-142-3p was down-regulated in breast cancer, which can negatively regulate the expression of CXCL12 and affect the expression of β-catenin, a key protein in the WNT signaling pathway, thus inhibiting breast cancer cell metastasis, EMT and stemness. Our findings provide a new molecular mechanism explaining the metastasis and stemness of breast cancer, offering a new potential target for the development of targeted therapies for this disease.

## Materials and methods

### Clinical specimens

Tumor tissues and adjacent non-tumor tissues were collected from 10 breast cancer patients at the First Affiliated Hospital of Bengbu Medical University. Prior to the resection of tumor tissues, none of the patients underwent chemotherapy, radiotherapy, surgery, or any alternative treatments. All participants signed informed consent forms, and the study received approval from the Ethics Committee of Bengbu Medical University (2023/No.339). All experiments are conducted according to relevant guidelines and regulations.

### Cell culture

The normal human mammary epithelial cell line (MCF 10 A) and the human breast cancer cell line (MCF 7) were purchased from the Shanghai Cell Bank of the Chinese Academy of Sciences. They were cultured in specialized cell media and Dulbecco’s Modified Eagle Medium (DMEM) from Gibco, USA, supplemented with 10% Fetal Bovine Serum (FBS) from Gibco, USA, respectively. Paclitaxel-resistant MCF 7 (MCF 7/PTX) was purchased from Wan Muchun Biological of Nanjing and cultured in 1640 medium (Gibco, USA) containing 10% FBS. Human renal epithelial (HEK) cell line 293T was purchased from Shanghai Institute of Cell Science, Chinese Academy of Sciences (Shanghai, China) and cultured using 1640 containing 10% serum. All cell cultures were maintained in a 37 °C cell incubator containing 5% CO_2_.

### Cell transfection

According to the reagent instructions, miR-142-3p mimics, miR-142-3p inhibitor, and CXCL12 interference fragment (GenePharma, China) were transfected into MCF 7 cells using lipofectamine2000 (Invitrogen, CA). Lentivirus containing miR-142-3p fragment (GenePharma, China) and SOX2 interference fragment (GenePharma, China) were transfected into MCF 7/PTX cells. After transfection for 24–48h, total RNA and proteins in the cells were extracted, and the transfection efficiency was verified by qRT-PCR and western blot. MCF 7 cells were treated with a WNT/β-catenin pathway inhibitor (ICG001, Beyotime, China) at a concentration of 5µM .

### RNA extraction and qRT-PCR

Total RNA from tissues (about 50 mg of tissue samples were cut and put into a tube equipped with magnetic beads, 1 ml Trizol was added to the tube, and placed in a cryogenic tissue grinder until ground into a homogenized state) and cells was extracted using Trizol reagent (Invitrogen, Carlsbad, CA), and its concentration was measured using a spectrophotometer (Thermo Scientific). The reverse transcription of mRNA into cDNA was carried out with Hiscript III RT supermix for qPCR (Vazyme, Nanjing, China). The expression levels of *CXCL12* were quantified using the All-in-One™ qPCR Mix (Genecopoeia, Guangzhou, China), with *GAPDH* serving as the internal reference gene. For miRNA, the All-in-all™ miRNA qRT-PCR Detection Kit 2.0 (Genecopoeia, Guangzhou, China) was utilized for reverse transcription, employing U6 as the internal reference to quantify miR-142-3p levels in tissues and cells. The relative RNA expression was determined using the 2^-ΔΔCt^ method. The nucleic acid sequences of the primers used are shown in Table 1.

**Table 1:** Primers used for Real-Time PCR analysis.

Gene	Sequences
U6	Forward: 5′-CTCGCTTCGGCAGCACA-3′ Reverse: 5′-AACGCTTCACGAATTTGCGT-3′
GAPDH	Forward: 5′ -CAGCCTCAAGATCATCAGCA − 3′ Reverse: 5′-TGTGGTCATGAGTCCTTCCA-3′
miR-142-3p	Forward: 5′-TGCGGTGTAGTGTTTCCTACTT-3′ Reverse: 5′-CCAGTGCAGGGTCCGAGGT-3′
CXCL12	Forward: 5′-CGUCAAGCAUCUCAAAAUUTT-3′ Reverse: 5′- AAUUUUGAGAUGCUUGACGTT-3′

### Western blot

The collected cells were lysed by adding an appropriate volume of cell lysate solution (RIPA: PMSF = 99:1) and incubated on ice for 30 min. This mixture was then centrifuged at 12,000 rpm for 15 min to separate the proteins. The BCA Protein Assay Kit (Beyotime, China) was employed to measure the protein concentration. Protein electrophoresis was performed using 10% and 12.5% SDS-PAGE gels (EpiZyme, China), followed by transfer onto PVDF membranes (Millipore, USA). The membranes were blocked with blocking solution (Beyotime, China) for 2 h and then washed with TBST. They were incubated overnight at 4 °C with primary antibodies (Table 2). The following day, after additional washes with TBST, the membranes were incubated with corresponding secondary antibodies (Proteintech, USA) at 37 °C for 2 h. The protein signals were detected using an ECL detection system (Millipore, USA).

**Table 2:** The primary antibody information used in the Western blot experiment.

Primary antibody	Source	Item number	Dilution ratio
GAPDH	Proteintech	10494-1-AP	1:5000
NANOG	Proteintech	14295-1-AP	1:1000
OCT4	Proteintech	11263-1-AP	1:1000
SOX2	Proteintech	11064-1-AP	1:1000
Snail	Allinifity	AF6032	1:1000
E-cadherin	Proteintech	20874-1-AP	1:1000
CXCL12	Proteintech	17402-1-AP	1:1000
β-catenin	Proteintech	51067-2-AP	1:1000
Pg-p	Proteintech	22336-1-AP	1:1000
BCRP	Proteintech	27286-1-AP	1:1000

### Immunofluorescence

Inoculating approximately 5 × 10^4^ breast cancer cells into a 24-well plate. After 48 h, the cells were fixed with 4% paraformaldehyde and permeabilized with 0.25% Triton X-100 for 15 min. Following a PBS wash, the cells were blocked with 5% BSA for 30 min. Then, 40 µl of β-catenin antibody (dilution 1:100) was applied to the cells and left overnight. The following day, 40 µl of fluorescein isothiocyanate (FITC)-labeled secondary antibody (dilution 1:50) was added and the cells were incubated in a 37 °C water bath in the dark for 30 min. After another PBS wash, 40 µl of 0.5 µg/ml DAPI (Beyotime, China) was added to each well for nuclear staining and incubated in the dark for 5 min. Finally, the cells were visualized and photographed using an inverted fluorescence microscope (Zeiss Obsever Z1, Germany).

### Microsphere formation assay

According to the experimental groups, 1 × 10^4^ cells of breast cancer cells were collected and inoculated in 6-well ultra-low attachment plate (Corning, China). Every 2–3 days, 500 µl of serum-free DMEM/F12 medium supplemented with 0.4% bovine serum albumin (BSA), 1% penicillin/streptomycin, 5 mg/L insulin, 20ng/ml epidermal growth factor (EGF), and 20ng/ml basic fibroblast growth factor (b-FGF) was added to each well. The cells were cultured for 14 days, after which they were observed and photographed under a microscope (Olympus CKX41, Japan) at 40× magnification. The number of microspheres exceeding 50 μm in diameter was recorded.

### Cell counting kit-8 (CCK-8) assay

The breast cancer cells were transfected with miR-142-3p, and cells were collected 24 h later and inoculated into 96-well plates with 5 × 10^3^ cells per well. To each well, 10µL of CCK-8 reagent and 100 µl of DMEM were added, followed by incubation at 37 °C for 1 h. The absorbance at 450 nm was measured using a microplate reader to assess cell proliferation. This process was repeated every 24 h, with cell proliferation being measured a total of three times.

### Plate cloning experiment

The breast cancer cells were digested and seeded into a 6-well plate at a density of 2 × 10^4^ cells per well. The medium was replaced every two days until the colonies were visible to the naked eye. Cells in each well were fixed with 700 µl of paraformaldehyde for 20 min. Subsequently, the excess liquid was discarded, and the wells were allowed to dry at room temperature. Next, 700 µl of crystal violet stain was added to each well, allowing the cells to dye for 30 min. After staining, the excess dye was washed away with PBS. The number of clones was counted by Image J software, and the clone formation rate was calculated (clone formation rate = (clone number/number of inoculated cells) /100%).

### Wound healing experiment

Once the breast cancer cells density in the 6-well plate reached 90%, a wound was created across the cell monolayer using the tip of a 10 µl pipette, applying uniform pressure. The dislodged cells were removed by washing with PBS. Cell migration into the wound area was documented through photographs taken at 0, 24, and 48 h intervals with an inverted microscope (Olympus CKX41, Japan). The mobility of the cells was quantified using ImageJ software.

### Transwell experiment

According to the experimental group, 24 h after transfection, breast cancer cells were digested with trypsin. Then, a 200 µl aliquot of serum-free DMEM cell suspension containing 8 × 10^3^ cells per well was placed in the upper chamber of the transwell. Beneath the transwell chamber, 800 µl of DMEM supplemented with 10% FBS was added and the setup was cultured for 24 h. After incubation, the old medium in the upper chamber was removed, and the cells were fixed with 800 µl of 4% paraformaldehyde for 20 min. Subsequently, the cells were stained with 800 µl of crystal violet for 30 min, followed by washing three times with PBS, drying, and photographed under a 40x inverted microscope (Olympus CKX41, Japan).

### Determination of apoptosis

The transfected MCF 7 cells were collected and washed twice with pre-cooled PBS. Each group of cells was divided into four tubes (without dye, single-stained FITC, single-stained PI, and double-stained FITC and PI groups), with approximately 5 × 10^5^ cells in each tube. Add 500 µl of 1x Annexin V binding solution to the cell suspension, and then add 5µl each of FITC or (and) PI fluorescent dye (Keygen Biotechnology, China). After staining at room temperature in the dark for 15 min, the cells were filtered using a flow cytometry filter to obtain a single-cell suspension. The apoptosis rate of the cells was evaluated by flow cytometry (Beckman, American).

### Dual luciferase reporter assay

By consulting the Starbase database, it was found that miR-142-3p possesses binding sites with CXCL12. Based on this information, plasmids including miR-142-3p NC (negative control), miR-142-3p mimics, wild-type CXCL12, and mutant CXCL12 were constructed in accordance with the binding site sequences, provided by Genechem, China. For the experimental setup, these plasmids were transfected into 293T cells, followed by change of medium 6 h post-transfection. 48 h after the transfection, firefly and Renilla luciferase activities were detected using a dual luciferase reporter assay kit (Promega, USA) on a multifunctional microplate reader (PerkinElmer Ensight, USA).

### Statistical analysis

For each experiment, three independent replicates were conducted. The statistical analysis was carried out using GraphPad Prism 8.0 software, employing the two-group T test or three-group variance analysis (ANOVA) as appropriate. A p-value of less than 0.05 was deemed to indicate statistical significance. Each experiment was independently repeated three times, with significance levels denoted as follows: *p < 0.05, **p < 0.01, ***p < 0.001, ****p < 0.0001.

## Results

### miR-142-3p is lower expressed in breast cancer tissue and MCF 7 cells

**Fig. 1:**
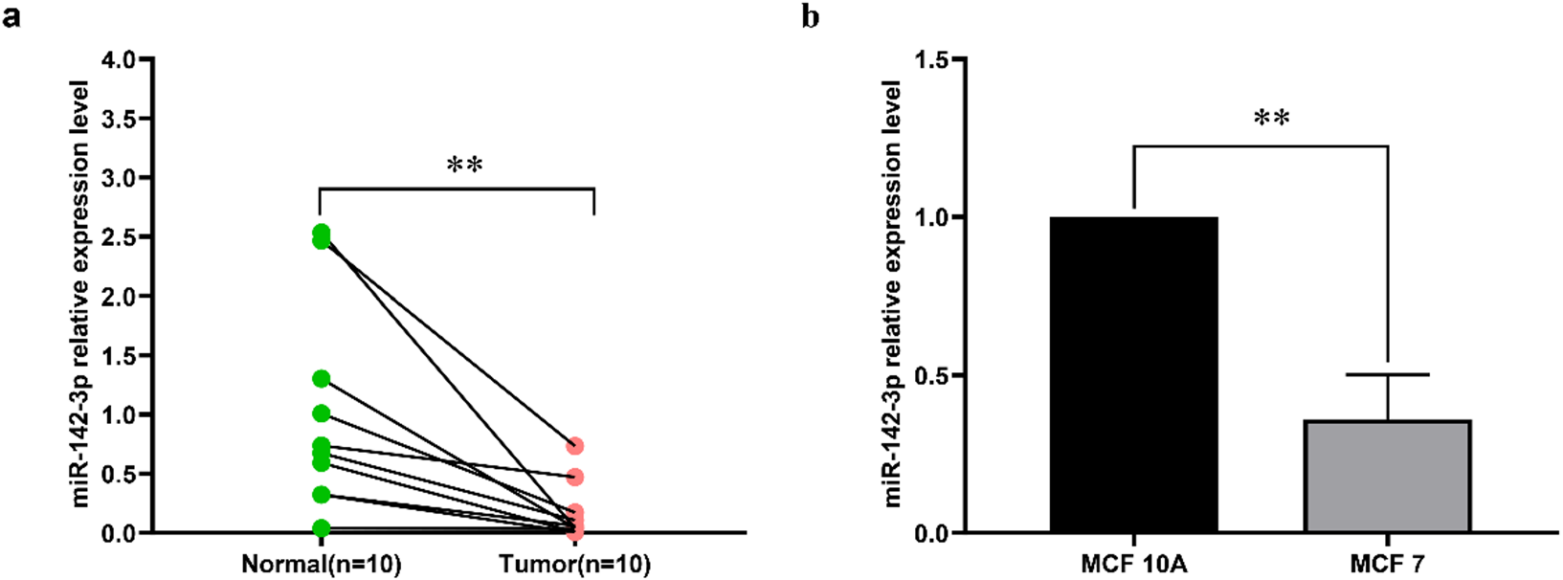
MiR-142-3p differential expression analysis (**a**) qRT-PCR was employed to detect the differential expression of miR-142-3p in the breast cancer and normal adjacent tissues. (**b**) qRT-PCR was used to detect the differential expression of miR-142-3p in MCF 10 A cells and MCF 7 cells. (**P < 0.01).

In this study, we collected breast cancer tissue samples and their adjacent normal tissue samples from 10 patients, and used qRT-PCR to determine the expression differences of miR-142-3p.Our findings indicate the that the expression of miR-142-3p is downregulated in breast cancer tissues compared to adjacent non-cancerous tissues ).(Fig. 1a). Additionally, the expression of miR-142-3p is significantly lower in MCF-7 cells compared to MCF-10 A cells. (Fig. 1b).

### The stemness of MCF 7 cells was higher than that of MCF 10 A cells

**Fig. 2:**
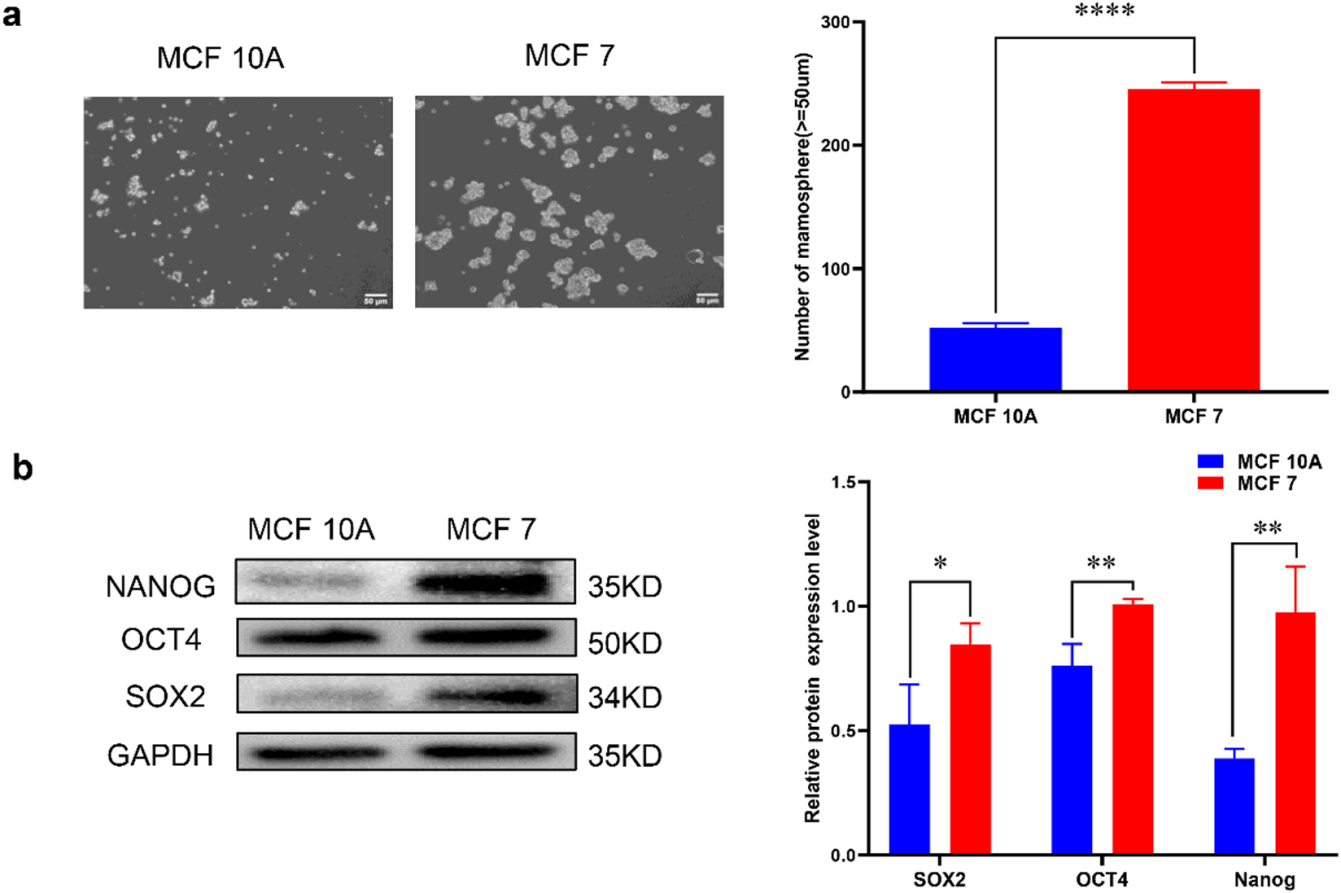
Comparison of stemness between MCF 10 A and MCF 7 (**a**) The microsphere formation experiment was conducted to observe the sphere-forming ability of both MCF 7 and MCF 10 A cells. (**b**) Protein expression of stemness markers in MCF 7 and MCF 10 A cells was examined using Western blot. (*P < 0.05, **P < 0.01, ****P < 0.0001).

Compared to MCF 10 A cells, MCF 7 cells have a greater tendency to form microspheres and are larger in volume in ultra-low attachment 6-well plates (Fig. 2a). Western blot experiments show elevated levels of markers associated with breast cancer stemness, including SOX2, OCT4, and NANOG (Fig. 2b). These results indicate that the stemness characteristics of MCF 7 cells surpass those of MCF 10 A cells.

### miR-142-3p inhibited the proliferation, migration, EMT and stemness of MCF 7 cells

**Fig. 3:**
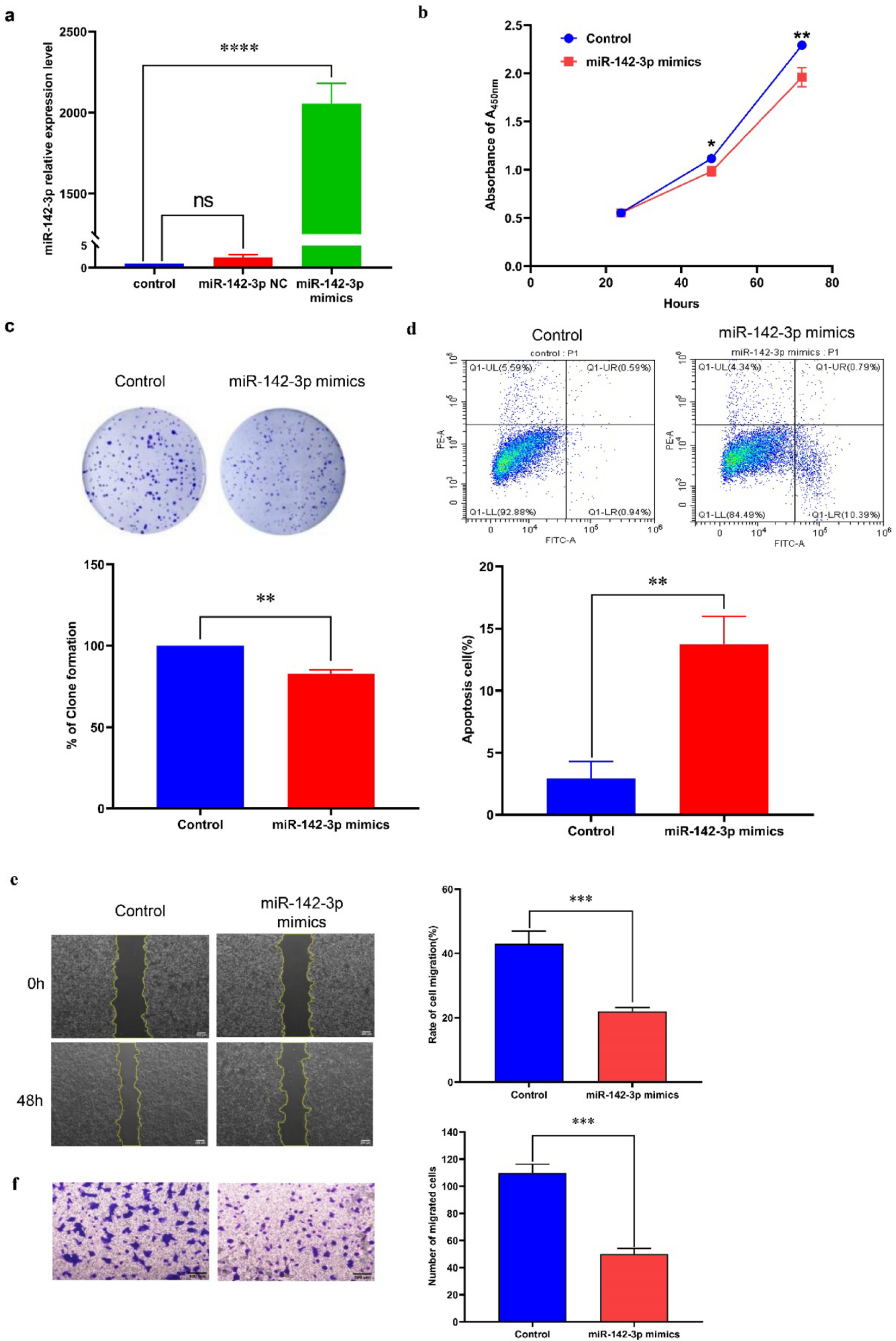

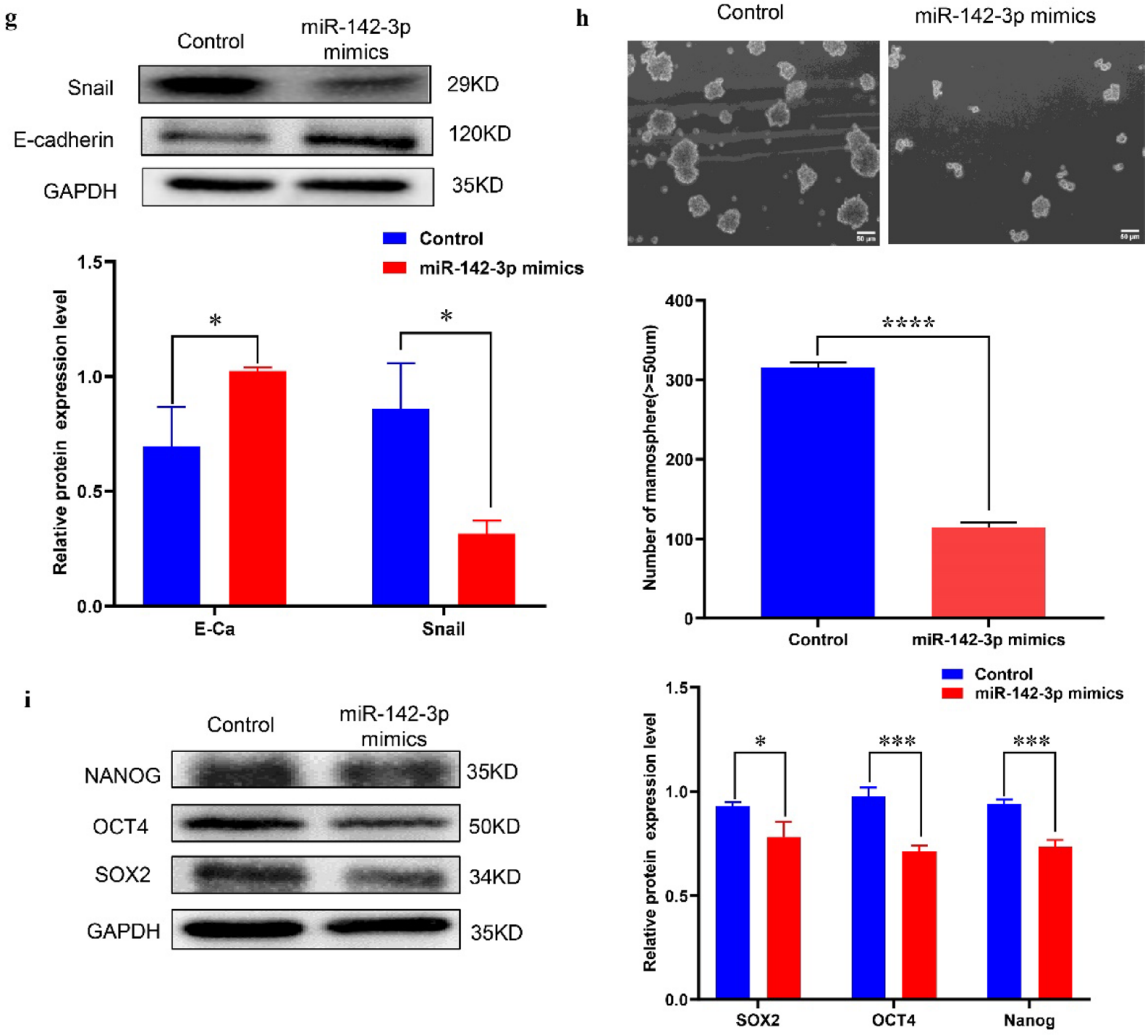
MiR-142-3p inhibits the proliferation, migration, EMT and stemness of breast cancer cells (**a**) The transfection efficiency of miR-142-3p mimics was assessed using qRT-PCR. (**b-c**) The impact of miR-142-3p on MCF 7 cell proliferation was examined through CCK8 and plate cloning experiments. (**d**) Flow cytometry was employed to investigate the regulatory role of miR-142-3p on apoptosis in MCF 7 cells. (**e-f**) The influence of miR-142-3p on MCF 7 cell migration was evaluated using wound healing and transwell assays. (**g**) Western blot was performed to analyze the effect of miR-142-3p on the regulation of EMT-related proteins. (**h**) Microsphere formation experiments were conducted to observe the effect of miR-142-3p on the spheroid formation ability of MCF 7 cells. (**i**) Western blot analysis was employed to assess the impact of miR-142-3p on the expression of stemness marker proteins. (*P < 0.05, **P < 0.01, ***P < 0.001, ****P < 0.0001).

To study the effect of miR-142-3p on the biological activity of breast cancer cells, we used Lipofectamine 2000 to transfect MCF 7 cells with miR-142-3p mimics. The results showed a significant increase in miR-142-3p content within the cells (Fig. 3a).Compared to the untreated group, miR-142-3p inhibited the proliferation of MCF 7 cells, which was confirmed by CCK8, plate cloning, and flow cytometry experiments (Fig. 3b-c) while also promoting apoptosis (Fig. 3d). Moreover, scratch and transwell experiments indicated that miR-142-3p mimics suppressed the migration of MCF 7 cells (Fig. 3e-f). Western blot analysis of proteins associated with epithelial-mesenchymal transition (EMT) revealed that miR-142-3p suppressed Snail protein expression and promoted E-cadherin protein expression (Fig. 3g). To assess the effect of miR-142-3p on the stemness of MCF 7 cells, cells transfected with miR-142-3p mimics were cultured in a specialized medium for microsphere formation 24 h post-transfection. After 14 days, a reduction in both the number and volume of microspheres was observed in the miR-142-3p mimics group compared to the control group (Fig. 3h). Additionally, Western blot experiments revealed a suppression in the expression of stemness-related markers (SOX2, OCT4, and NANOG) by miR-142-3p mimics (Fig. 3i). These results collectively indicated that miR-142-3p inhibits the stemness of breast cancer cells.

### MiR-142-3p can regulate the expression of CXCL12 in a targeted way

**Fig. 4:**
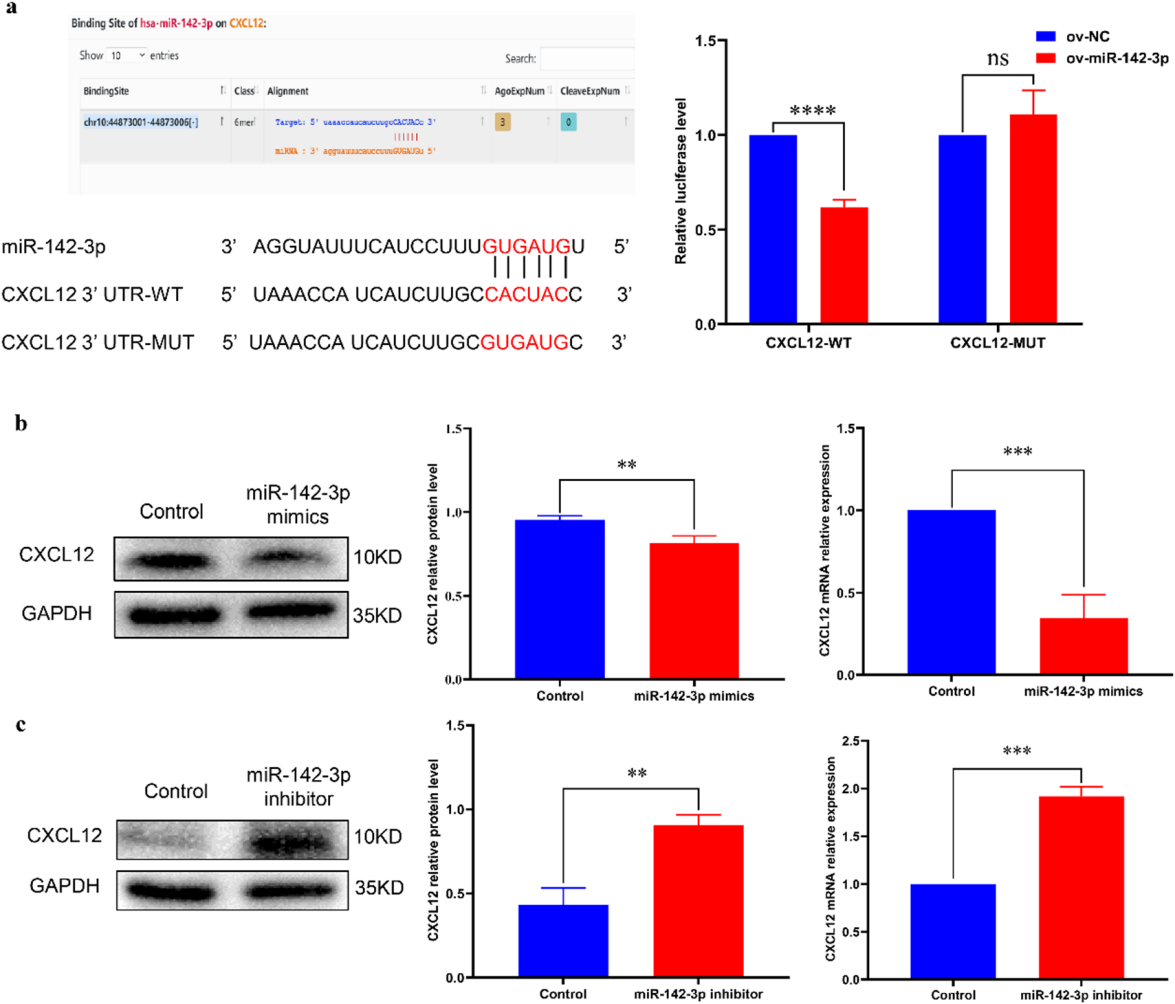
Verify the targeted regulatory between miR-142-3p and CXCL12. (**a**) Starbase database and dual luciferase reporter assays were employed to identify the binding site between miR-142-3p and CXCL12. (**b**) The impact of miR-142-3p mimics on the protein and mRNA expression levels of CXCL12 was assessed. (**c**) MiR-142-3p inhibitor can affect both CXCL12 protein and mRNA expression. (**P < 0.01, ***P < 0.001, ****P < 0.0001).

Through bioinformatics database prediction, CXCL12 and miR-142-3p were found to have binding sites. Experimental analysis using dual luciferase reporter assays showed that overexpression of miR-142-3p decreased the luciferase activity in 293T cells transfected with CXCL12-WT plasmid, but had no effect on transfected with CXCL12-MUT plasmid. Therefore, we determined targeting relationship between miR-142-3p and CXCL12 (Fig. 4a). Transfection of miR-142-3p mimics resulted in a significant reduction in both mRNA and protein expression levels of CXCL12 (Fig. 4b), whereas miR-142-3p inhibitors promoted mRNA and protein expression of CXCL12 (Fig. 4c). These experimental findings collectively indicate that the expression of CXCL12 is regulated by miR-142-3p targeting.

### MiR-142-3p can regulate the migration, EMT and stemness of MCF 7 cells by targeting CXCL12 expression

**Fig. 5:**
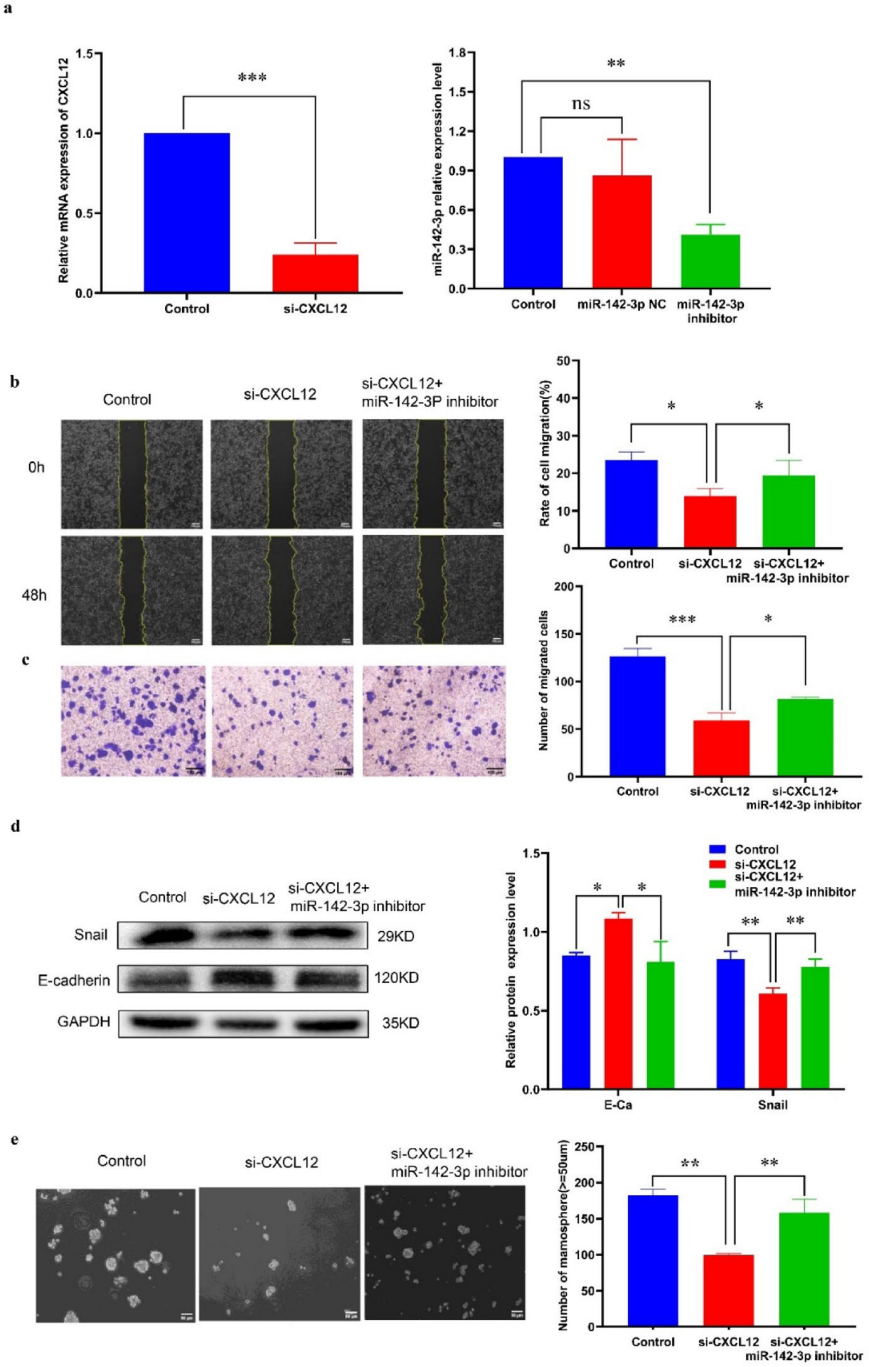

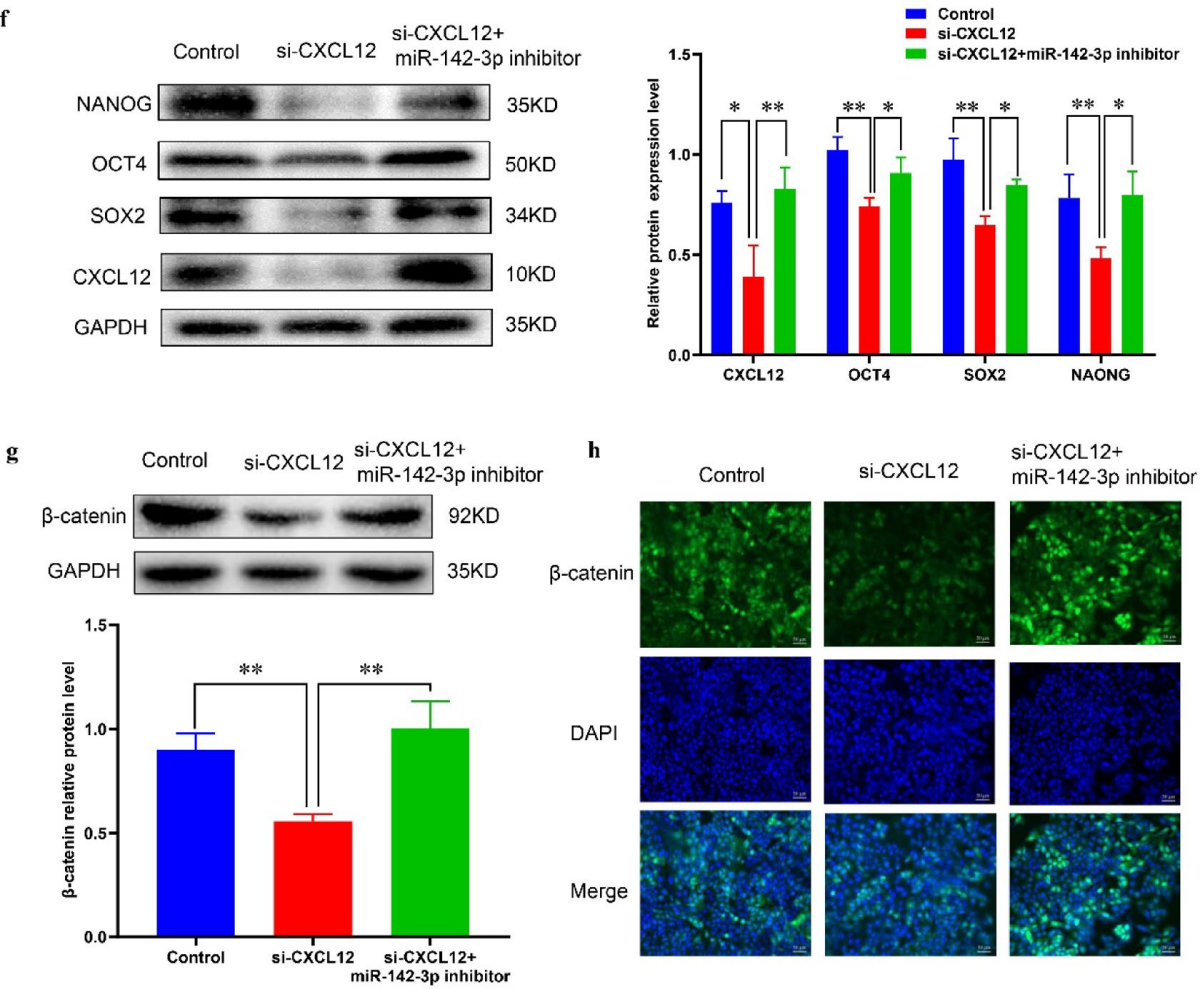
MiR-142-3p targets CXCL12 to regulate the migration, EMT and stemness of MCF 7 cells (**a**) The efficiency of CXCL12 siRNA and miR-142-3p inhibitor transfection was assessed using qRT-PCR. (**b-c**) The migratory capacity of MCF 7 cells across various treatment groups was compared using wound healing and transwell assays. (**d**) Western blot results exhibited distinct levels of EMT-related proteins among different experimental groups. (**e**) The microsphere formation ability of MCF 7 cells in different treatment groups was compared. (**f**) The expression levels of SOX2, OCT4, and NANOG proteins in different experimental groups were determined by Western blot. (**g**) Western blot analysis was conducted to examine the impact of miR-142-3p and CXCL12 on the expression of β-catenin protein. (**h**) The influence of miR-142-3p and CXCL12 on the expression of β-catenin protein was investigated through immunofluorescence experiments. (*P < 0.05, **P < 0.01, ***P < 0.001).

To further investigate the potential mechanism by which miR-142-3p targets CXCL12 in regulating stem cell characteristics, we inhibited the expression of both CXCL12 and miR-142-3p in MCF 7 cells. The results showed that the expression of both CXCL12 and miR-142-3p was significantly inhibited(Fig. 5a). After interference with CXCL12, scratch and transwell assays indicated a decrease in cell migration rate and a decrease in the number of migrating cells. Conversely, inhibition of miR-142-3p reversed these effects (Fig. 5b-c). Western blot experiments also showed that interference with CXCL12 expression led to an increase in E-cadherin protein expression and a decrease in Snail protein expression, indicating that the EMT capability was suppressed. Interestingly, this result was also reversed by the miR-142-3p inhibitor (Fig. 5d). The results of microsphere formation experiment showed that interference with CXCL12 resulted in a reduction of both the number and volume of microsphere formation, and this inhibition was counteracted by the miR-142-3p inhibitor (Fig. 5e). Furthermore, Western blot analysis showed that the expression of stem cell-related proteins decreased after CXCL12 interference, however, the miR-142-3p inhibitor mitigated this effect (Fig. 5f). Based on these findings,, interference with CXCL12 can inhibit the migration, EMT and stemness of breast cancer cells, and miR-142-3p inhibitor can attenuate this inhibitory effect. These results suggest that miR-142-3p targeting CXCL12 plays a synergistic regulatory role of in modulating the migration, EMT and stemness of breast cancer cells.

During this process, we observed that the level of β-catenin was modulated by both miR-142-3p and CXCL12. Western blot experiments showed that CXCL12 siRNA suppressed the expression of β-catenin protein, and this inhibition was attenuated by the miR-142-3p inhibitor (Fig. 5g). Immunofluorescence results also depicted the alteration in β-catenin expression during this process (Fig. 5h).

### MiR-142-3p can regulate the migration, EMT and stemness of MCF 7 cells through modulating the WNT/β-catenin pathway

**Fig. 6:**
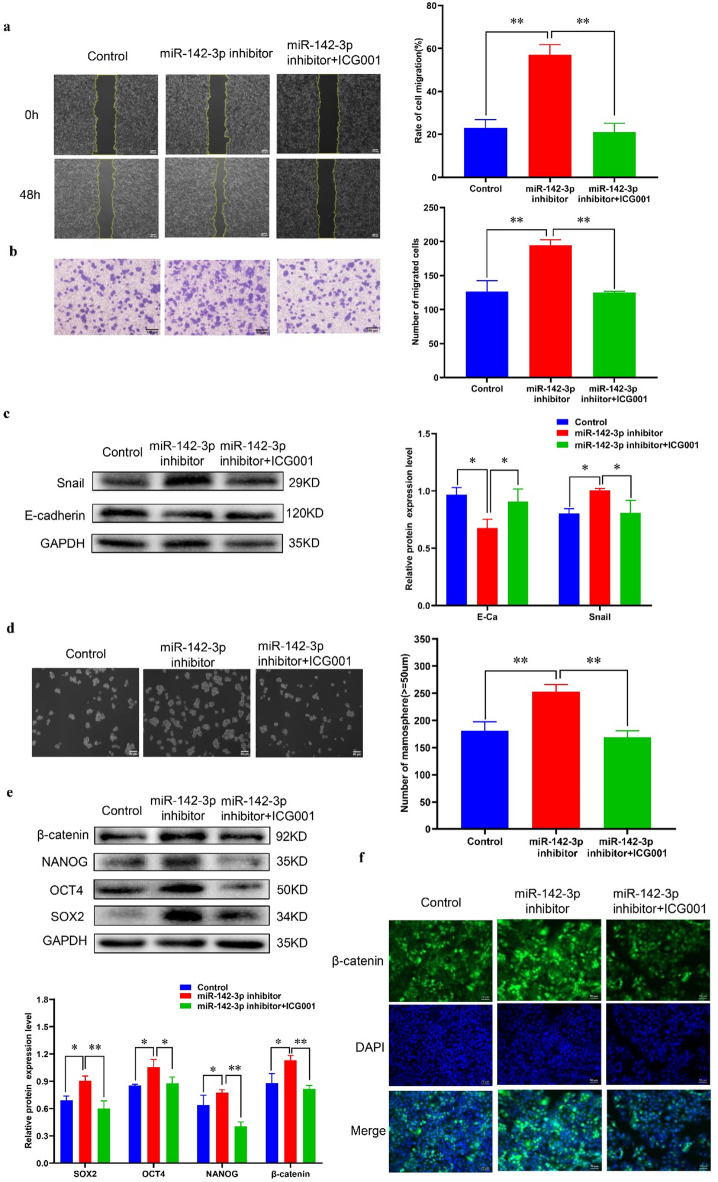
MiR-142-3p can regulate the migration, EMT and stemness of MCF 7 through affecting the WNT/β-catenin signaling pathway (**a-b**) The impact of various treatments on the migratory capability of MCF 7 cells was assessed using scratch and transwell assays. (**c**) Western blot analysis was conducted to examine the expression levels of E-cadherin protein and Snail protein across different treatment groups; (**d**) The effects of different treatments on the stemness of MCF 7 cells were observed through microsphere formation experiments; (**e**) Western blot analysis was utilized to investigate the regulatory effects of miR-142-3p inhibitor and ICG001 on the expression of stemness proteins in MCF 7 cells; (**f**) Immunofluorescence assays were employed to evaluate the effects of different treatments on the expression of β-catenin protein in MCF 7 cells. (*P < 0.05, **P < 0.01).

An increasing number of studies indicate that the WNT signaling pathway relies on β-catenin and T-cell factor (TCF)/lymphoid enhancer factor (LEF), plays a crucial role in breast cancer cell proliferation and the maintenance of stemness^17^. Therefore, in this study, we aimed to explore the involvement of the WNT/β-catenin signaling pathway in regulating MCF 7 cell stemness by miR-142-3p. To achieve this objective, we utilized the WNT/β-catenin inhibitor ICG001. Scratch and transwell experiments demonstrated that miR-142-3p influenced the migration of MCF 7 cells through the WNT/β-catenin signaling pathway (Fig. 6a-b). Western blot analysis revealed that inhibition of miR-142-3p led to decreased E-cadherin protein expression and increased Snail protein expression, which could be reversed by ICG001. This suggests that miR-142-3p influences the EMT process of breast cancer cells via the WNT/β-catenin signaling pathway (Fig. 6c). The results of the microsphere formation experiment indicated that inhibition of miR-142-3p enhanced both the number and volume of microsphere formation in MCF 7 cells, whereas this enhancement was significantly uppressed by the addition of ICG001 (Fig. 6d). Additionally, Western blot analysis indicated that the miR-142-3p inhibitor promoted the expression of stemness marker proteins and β-catenin, an effect that could be blocked by ICG001 (Fig. 6e). Immunofluorescence assays further supported that miR-142-3p regulates the WNT/β-catenin signaling pathway by regulating the expression of β-catenin protein. (Fig. 6f).

### MiR-142-3p inhibited the drug resistance of MCF 7/PTX cells

**Fig. 7:**
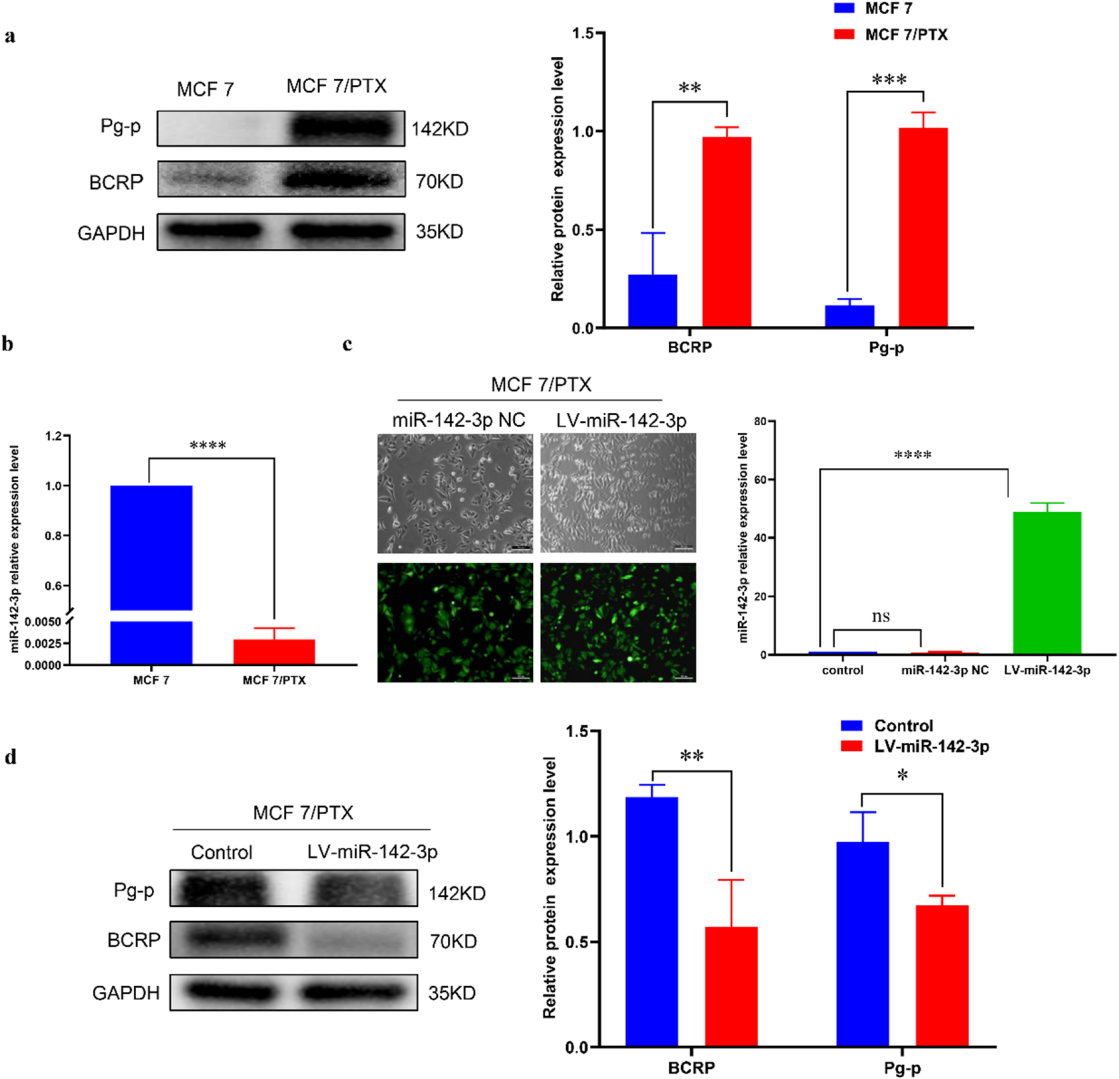
miR-142-3p can inhibit the drug resistance of MCF 7/PTX cells (**a**) Western blot analysis was conducted to confirm the drug resistance status of MCF 7/PTX cells. (**b**) Differential expression of miR-142-3p in MCF 7/PTX cells and MCF 7 cells was verified using qRT-PCR. (**c**) The transfection efficiency of miR-142-3p lentivirus was assessed through immunofluorescence and qRT-PCR experiments. (**d**) Western blot analysis was performed to investigate the impact of miR-142-3p on the expression of drug-resistant proteins. (*P < 0.05, **P < 0.01, ***P < 0.001, ****P < 0.0001).

Paclitaxel is widely used microtubule stabilizer in chemotherapy, however, drug resistance frequently leads to treatment failure in breast cancer patients. P-glycoprotein (P-gp) and breast cancer resistance protein (BCRP) which promote the efflux anti-tumor drugs from intracellular to extracellular compartments, is associated with the development of chemoresistance in cancer cells. To decipher the mechanismof drug resistance, we focused on the paclitaxel-resistant breast cancer cell line MCF 7/PTX. Western blot experiments revealed that expression of drug resistance proteins (BCRP, Pg-p) was significantly elevated in MCF 7/PTX cells compared to MCF 7 cells (Fig. 7a). We further explored the expression of miR-142-3p in MCF 7/PTX cells, and found that its expression was significantly lower in comparison to MCF 7 cells (Fig. 7b). Consequently, we stably transfected miR-142-3p lentivirus into MCF 7/PTX cells and confirmed successful transfection under a fluorescence microscope. qRT-PCR analysis confirmed the successful establishment of MCF 7/PTX cells with stable overexpression of miR-142-3p lentivirus (Figure.7c). Subsequent Western blot experiments demonstrated that miR-142-3p inhibited the expression of drug-resistant proteins in MCF 7/PTX cells (Fig. 7d), suggesting its role in attenuating drug resistance in breast cancer cells.

### SOX2 can regulate the drug resistance, proliferation, migration and EMT of MCF 7/PTX

**Fig. 8:**
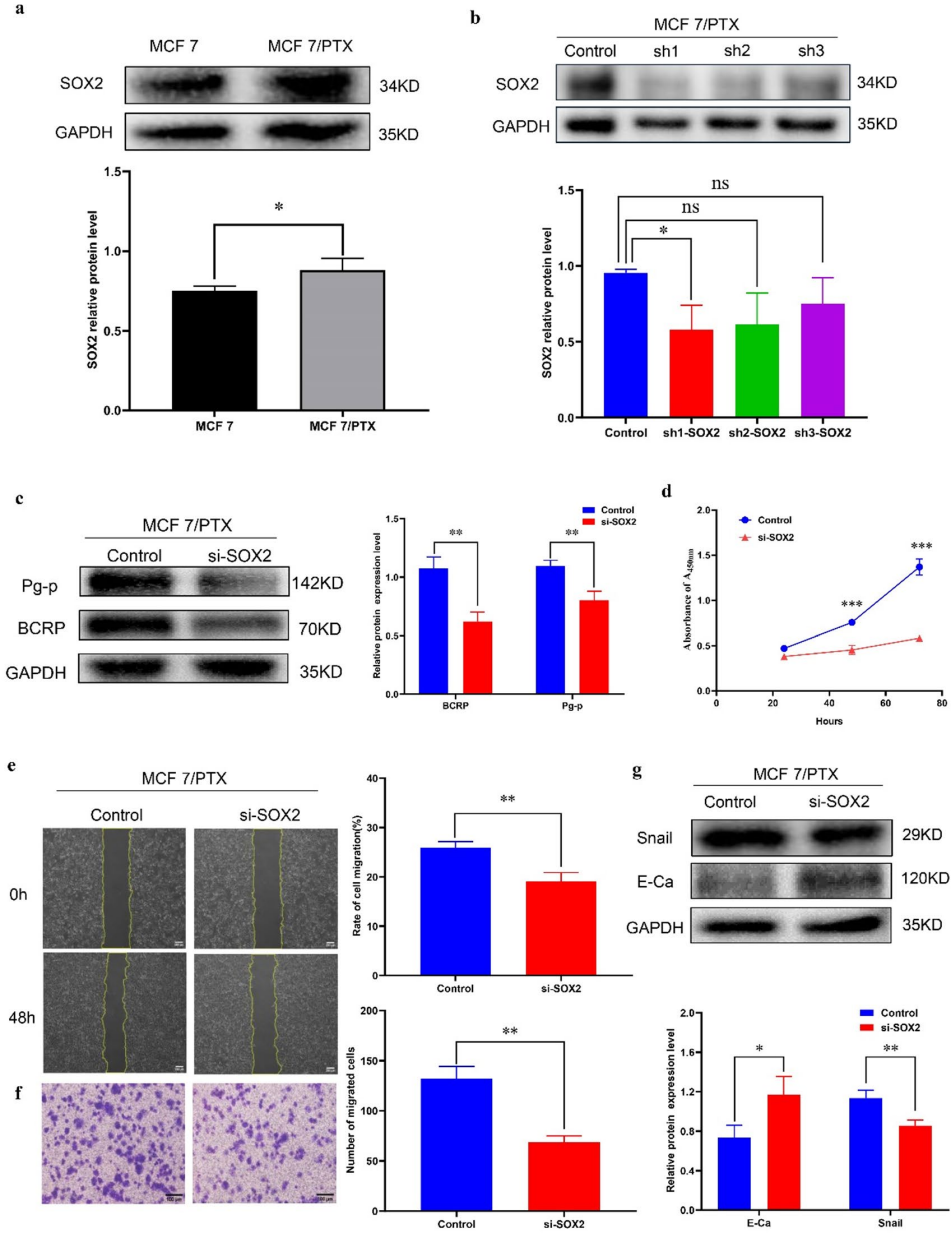
SOX2 can regulate the drug resistance, proliferation, migration and EMT of MCF 7/PTX cells (**a**) The expression of SOX2 protein in MCF 7/PTX cells and MCF 7 cells was examined using Western blot analysis. (**b**) Western blot experiment was conducted to compare the effects of three interference fragments (sh1, sh2, sh3). (**c**) Western blot analysis was employed to assess the impact of SOX2 on the levels of drug resistance proteins in MCF 7/PTX cells. (**d**) The effects of SOX2 on the proliferation of MCF 7/PTX cells were evaluated using CCK8 assays. (**e-f**) The influence of SOX2 on the migration of MCF 7/PTX cells was assessed through wound healing and transwell assays. (**g**) Western blot analysis was conducted to investigate the effect of SOX2 on the expression of EMT-related proteins. (*P < 0.05, **P < 0.01, ***P < 0.001).

As a transcription factor associated with stemness, SOX2 has been implicated in promoting cancer progression in various studies. Building upon our previous experimental findings revealing the inhibitory effect of miR-142-3p on SOX2 expression via the CXCL12/WNT/β-catenin signaling pathway, we observed elevated expression of SOX2 in MCF 7/PTX cells compared to MCF 7 cell (Fig. 8a). To further explore the role of SOX2 in the development of breast cancer, we transfected three SOX2 interference fragments (sh1, sh2, sh3) into MCF 7/PTX cells respectively. Through western blot experiments, it was found that sh1 was the most effective interference fragment (Fig. 8b). Compared with the control group, interference with SOX2 reduced the expression level of drug-resistant protein (BCRP, Pg-p) in MCF7/PTX cells, indicating that SOX2 has the effect of increasing taxol resistance (Fig. 8c). Moreover, experiments revealed a decrease in the proliferation capacity of MCF 7/PTX cells (Fig. 8d), accompanied by reduced migration and EMT abilities (Fig. 8e-g). These findings collectively suggest a role for SOX2 in promoting drug resistance and cancer progression in breast cancer cells.

## Discussion

Metastasis significantly contributes to increased mortality among cancer patients. The EMT is process is not only closely related to cancer metastasis but also associated wit cancer drug resistance and stemness^2^. Studies have found that EMT can enhance the ability of breast epithelial cells to form microspheres and express higher levels of stem cell markers, underlining the deep connection between EMT and cancer stemness^18^. A study featured in *Nature* highlighted the role of RHOJ, a small GTPase in cancer-associated EMT, showing its capability to counteract chemotherapy resistance by swiftly repairing chemotherapy-induced DNA damage^19^. Unlike cancer cells, CSCs consume less glucose and produce less lactose^20^. BCSCs can also secrete DKK1 to promote the expression of SLC7A11, thereby protecting cancer cells from ferroptosis^21^. At the same time, CSCs can also interact with immune cells in the TME by attracting myeloid-derived suppressor cells (MDSCs) to inhibit the immune response against the tumor^22^. These mechanisms collectively enhance the resilience of CSCs. Given the critical role CSCs in tumor progression, targeting them for elimination or preventing their differentiation has become aprimary objective in cancer therapy. Deepening our understanding of the molecular and biochemical traits of CSCs will pave the way fordeveloping novel, targeted treatments aimed at CSC eradication.

Increasing evidence highlights the pivotal role of miRNA in modulating EMT, cancer stemness and drug resistance^23^. Specifically, the upregulation of miR-221/222 in breast cancer leads to the suppression of PTEN, enhances phosphorylation of Akt, increases the proportion of CD44^+^CD24^-^ cell subsets, and improves the ability of cancer cells to form microsphere formation, indicating that miR-221/222 can promote the self-renewal of BCSCs^24^. Similarly overexpression of miR-128-3p can effectively disrupts the WNT signaling pathway and diminish BCSC-like traits by down-regulating the expression of NEK2^25^. Moreover, in some studies, miR-142-3p has been reported to be associated cancer stemness. For example, Huang et al. identified miR-142-3p as a inhibitor of colorectal cancer growth and stem cell properties^26^. Although the association between miR-142-3p and breast cancer nhibition has been recognized^27^, its influence on EMT and stemness in breast cancer has not been extensively studied. This study first discovered that miR-142-3p can affect migration, EMT and stemness of breast cancer cells by specifically targeting and regulating CXCL12 expression. Gupta and colleagues used the Markov model to predict the transformation of non-stem cell-like cells into breast cancer stem cell-like cells. Their experimental evidence demonstrated that even tumor tissues initially lacking CSCs, have the capacity to regenerate CSCs at the transplantation site after a sufficient period of time^28^. This suggests that breast cancer cells exhibiting a stem cell-like phenotype have the potential to evolve into BCSCs, thereby promoting the progression of breast cancer.

**Fig. 9:**
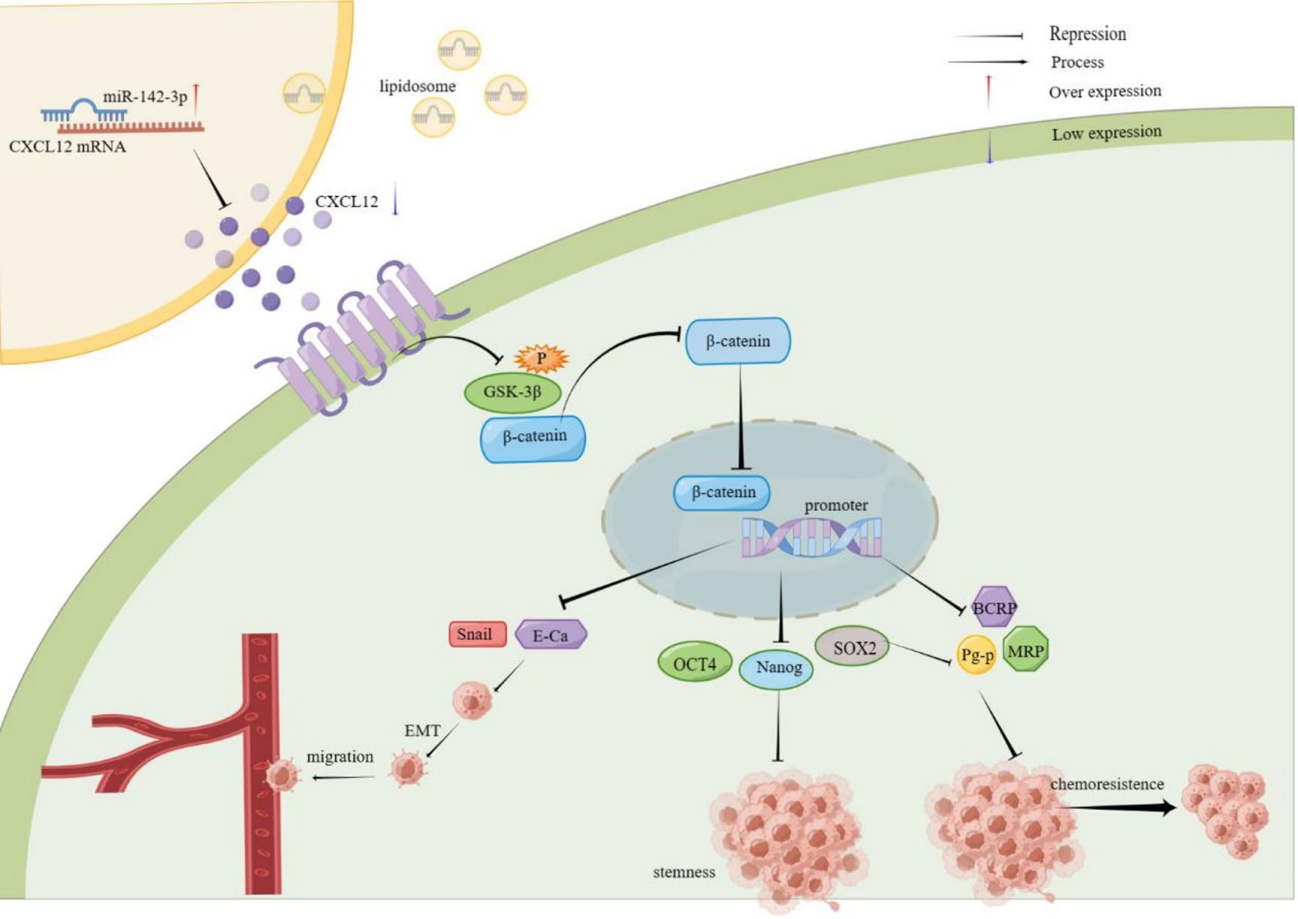
MiR-142-3p targets the CXCL12/WNT/β-catenin signaling pathway to regulate the stemness, EMT and drug resistance of breast cancer cells.

The metastasis of tumor cells through the EMT process and the maintenance of a stemness phenotype are influenced by a multitude of factors. Several key intracellular signaling pathways including WNT, NF-κB, Notch, Hedgehog, JAK-STAT, and PI3K/Akt/mTOR have been identified as regulators of CSC growth^29^. Within the TME, the WNT signaling network plays a crucial role in directing cancer behaviors such as dormancy, resistance to therapy, EMT, immune evasion proliferation, and stemness maintenance. The classical WNT pathway functions through Frizzled and LRP5/6 receptors, signaling to β-catenin-TCF/LEF, which then increases the expression of genes like CCND1 and MYC to stimulate CSC proliferation. Non-canonical WNT signaling promotes CSC dormancy by modulating WNT signaling inhibition and interacting with other pathways such as TGF-β^30^. MiRNA can also contribute to this regulatory network, as seen in the regulation of the WNT signaling pathway. For instance, miR-192 and miR-215, found at elevated levels in gastric cancer, are known to activate the WNT/β-catenin pathway. This activation enhances the proliferation and migration of gastric cancer cells by suppressing APC expression^31^. Although our results indicate a suppressive role for miR-142-3p in various processes of breast cancer development, further investigation is required to elucidate its specific mechanism. In this study, we discovered that suppressing miR-142-3p led to an increase in β-catenin expression. This observation indicated that the WNT/β-catenin signaling pathway was activated, which also enhanced the migration, EMT, and stemness characteristics of breast cancer cells. Interestingly, after combined treatment with WNT/β-catenin inhibitor ICG001, the effect of miR-142-3p inhibitor was reversed. This suggests that miR-142-3p can play a crucial role in hindering breast cancer cell migration, EMT, and stemness by acting through the WNT/β-catenin signaling pathway, as illustrated in Fig. 9.

Paclitaxel is widely utilized in the clinical chemotherapy treatment of breast cancer. The protein SOX2, known for its role in maintaining stemness, has been closely linked to the drug resistance of cancer cells. Studies have showen that SOX2 can enhance the drug resistance of melanoma to paclitaxel by activating ABCC1^32^, and it has been shown to affect the responsiveness of lung cancer cells to osimotinib through the induction of autophagy^33^. Consequently, the effects ofof miR-142-3p and SOX2 on the migration, EMT, and paclitaxel resistance was examined in MCF 7/PTX cells. The results revealed that miR-142-3p inhibited the expression of drug resistance protein in MCF 7/PTX cells. Similarly, disrupting SOX2 expression also led to decreased levels of these drug resistance proteins. This indicates that miR-142-3p can control SOX2 expression in breast cancer cells via the CXCL12/WNT/β-catenin signaling pathway, thereby influencing their resistance to paclitaxel.

Currentt treatment strategies for BCSCs include natural compounds and their derivatives, antibody-based biopharmaceuticals, cell therapies, and small molecule inhibitors. However, th ecomplexity and dynamic nature of BCSCs, along with their complex interactions within the TME, presents substantial obstacles to effective targeted treatments^34^. Consequently, a promising avenue of therapy involves targeting the regulatory mechanisms that sustain the BCSC niche, in conjunction with conventional methods aimed at inhibiting cancer cell proliferation. This study highlights the role of miR-142-3p as a critical regulator of both the proliferation and the stemness characteristics of breast cancer cells, underscoring its potential utility as a clinical diagnostic biomarker and as an attractive therapeutic target.

## Conclusion

In this study, We observed that the expression level of miR-142-3p was downregulated in breast cancer. Our findings indicate that the upregulation of miR-142-3p can significantly hamper the progression of breast cancer. Specifically, miR-142-3p can negatively regulate CXCL12 expression and impede the WNT/β-catenin signaling pathway. This action collectively can suppress proliferation, migration, epithelial-mesenchymal transition (EMT), and stemness in breast cancer cells. Additionally, our research suggests that miR-142-3p could modulate breast cancer cell resistance to paclitaxel by downregulating SOX2 expression. These insights unveil potential targets for novel therapeutic strategies aimed at curbing breast cancer’s aggressiveness and improving patient outcomes.

## Supplementary Information

Below is the link to the electronic supplementary material.

Supplementary Material 1

## Data Availability

The datasets used and/or analysed during the current study available from the corresponding author on reasonable request.

## References

[1] Siegel, R. L. et al. Cancer statistics, 2023[J]. *CA Cancer J. Clin.* **73** (1), 17–48 (2023). 36633525 10.3322/caac.21763

[2] Hashemi, M. et al. EMT mechanism in breast cancer metastasis and drug resistance: revisiting molecular interactions and biological functions[J]. *Biomed. Pharmacother*. **155**, 113774. 10.1016/j.biopha.2022.113774 (2022). 36271556 10.1016/j.biopha.2022.113774

[3] Lee, K. L. et al. Triple-Negative breast cancer: current Understanding and future therapeutic breakthrough targeting cancer Stemness[J]. *Cancers (Basel)*. **11** (9). 10.3390/cancers11091334 (2019). 10.3390/cancers11091334 PMC6769912 31505803

[4] Kaufhold, S., Garban, H. & Bonavida, B. Yin Yang 1 is associated with cancer stem cell transcription factors (SOX2, OCT4, BMI1) and clinical implication[J]. *J. Exp. Clin. Cancer Res.* **35**, 84. 10.1186/s13046-016-0359-2 (2016). 27225481 10.1186/s13046-016-0359-2 PMC4881184

[5] Cao, M. et al. Cancer-cell-secreted extracellular vesicles suppress insulin secretion through miR-122 to impair systemic glucose homeostasis and contribute to tumour growth[J]. *Nat. Cell. Biol.* **24** (6), 954–967. 10.1038/s41556-022-00919-7 (2022). 35637408 10.1038/s41556-022-00919-7 PMC9233030

[6] Ji, H. et al. Two circPPFIA1s negatively regulate liver metastasis of colon cancer via miR-155-5p/CDX1 and HuR/RAB36[J]. *Mol. Cancer*. **21** (1), 197. 10.1186/s12943-022-01667-w (2022). 36224588 10.1186/s12943-022-01667-w PMC9555114

[7] Yang, Y. et al. Targeting the miR-34a/LRPPRC/MDR1 axis collapse the chemoresistance in P53 inactive colorectal cancer[J]. *Cell. Death Differ.* **29** (11), 2177–2189. 10.1038/s41418-022-01007-x (2022). 35484333 10.1038/s41418-022-01007-x PMC9613927

[8] Zou, Y. et al. Extracellular vesicles carrying miR-6836 derived from resistant tumor cells transfer cisplatin resistance of epithelial ovarian cancer via DLG2-YAP1 signaling pathway[J]. *Int. J. Biol. Sci.* **19** (10), 3099–3114. 10.7150/ijbs.83264 (2023). 37416779 10.7150/ijbs.83264 PMC10321283

[9] Shi, Y. et al. miR-142-3p improves Paclitaxel sensitivity in resistant breast cancer by inhibiting autophagy through the GNB2-AKT-mTOR Pathway[J]. *Cell. Signal.* **103**, 110566. 10.1016/j.cellsig.2022.110566 (2023). 36539001 10.1016/j.cellsig.2022.110566

[10] Li, J. et al. S100A9-CXCL12 activation in BRCA1-mutant breast cancer promotes an immunosuppressive microenvironment associated with resistance to immunotherapy[J]. *Nat. Commun.* **13** (1), 1481. 10.1038/s41467-022-29151-5 (2022). 35304461 10.1038/s41467-022-29151-5 PMC8933470

[11] Chen, S. et al. Cancer–associated fibroblast–induced M2–polarized macrophages promote hepatocellular carcinoma progression via the plasminogen activator inhibitor–1 pathway[J]. *Int. J. Oncol.* **59** (2). 10.3892/ijo.2021.5239 (2021). 10.3892/ijo.2021.5239 PMC8253588 34195849

[12] Feng, W. et al. CXCL12-mediated HOXB5 overexpression facilitates colorectal cancer metastasis through transactivating CXCR4 and ITGB3[J]. *Theranostics* **11** (6), 2612–2633. 10.7150/thno.52199 (2021). 33456563 10.7150/thno.52199 PMC7806482

[13] Wang, H. F. et al. The N-terminal polypeptide derived from vMIP-II exerts its antitumor activity in human breast cancer through CXCR4/miR-7-5p/Skp2 pathway[J]. *J. Cell. Physiol.* **235** (12), 9474–9486. 10.1002/jcp.29755 (2020). 32372405 10.1002/jcp.29755

[14] Wang, Y. et al. NT21MP negatively regulates paclitaxel-resistant cells by targeting miR–155–3p and miR–155-5p via the CXCR4 pathway in breast cancer[J]. *Int. J. Oncol.* **53** (3), 1043–1054. 10.3892/ijo.2018.4477 (2018). 30015868 10.3892/ijo.2018.4477 PMC6065429

[15] Yang, F. et al. Inhibition of dipeptidyl Peptidase-4 accelerates Epithelial-Mesenchymal transition and breast cancer metastasis via the CXCL12/CXCR4/mTOR Axis[J]. *Cancer Res.* **79** (4), 735–746. 10.1158/0008-5472.CAN-18-0620 (2019). 30584072 10.1158/0008-5472.CAN-18-0620

[16] Shi, X. et al. Exosome-derived miR-372-5p promotes stemness and metastatic ability of CRC cells by inducing macrophage polarization[J]. *Cell. Signal.* **111**, 110884. 10.1016/j.cellsig.2023.110884 (2023). 37690660 10.1016/j.cellsig.2023.110884

[17] Xu, X. et al. Wnt signaling in breast cancer: biological mechanisms, challenges and opportunities[J]. *Mol. Cancer*. **19** (1), 165. 10.1186/s12943-020-01276-5 (2020). 33234169 10.1186/s12943-020-01276-5 PMC7686704

[18] Mani, S. A. et al. The epithelial-mesenchymal transition generates cells with properties of stem cells[J]. *Cell* **133** (4), 704–715. 10.1016/j.cell.2008.03.027 (2008). 18485877 10.1016/j.cell.2008.03.027 PMC2728032

[19] Debaugnies, M. et al. RHOJ controls EMT-associated resistance to chemotherapy[J]. *Nature* **616** (7955), 168–175. 10.1038/s41586-023-05838-7 (2023). 36949199 10.1038/s41586-023-05838-7 PMC10076223

[20] Vlashi, E. et al. Metabolic state of glioma stem cells and nontumorigenic cells[J]. *Proc. Natl. Acad. Sci. U S A*. **108** (38), 16062–16067. 10.1073/pnas.1106704108 (2011). 21900605 10.1073/pnas.1106704108 PMC3179043

[21] Wu, M. et al. Cancer stem cell regulated phenotypic plasticity protects metastasized cancer cells from ferroptosis[J]. *Nat. Commun.* **13** (1), 1371. 10.1038/s41467-022-29018-9 (2022). 35296660 10.1038/s41467-022-29018-9 PMC8927306

[22] Bayik, D. & Lathia, J. D. Cancer stem cell-immune cell crosstalk in tumour progression[J]. *Nat. Rev. Cancer*. **21** (8), 526–536. 10.1038/s41568-021-00366-w (2021). 34103704 10.1038/s41568-021-00366-w PMC8740903

[23] Pan, G. et al. EMT-associated MicroRNAs and their roles in cancer stemness and drug resistance[J]. *Cancer Commun. (Lond)*. **41** (3), 199–217. 10.1002/cac2.12138 (2021). 33506604 10.1002/cac2.12138 PMC7968884

[24] Li, B. et al. miR-221/222 enhance the tumorigenicity of human breast cancer stem cells via modulation of PTEN/Akt pathway[J]. *Biomed. Pharmacother*. **79**, 93–101. 10.1016/j.biopha.2023 (2016). 27044817 10.1016/j.biopha.2016.01.045

[25] Chen, Y. et al. microRNA-128-3p overexpression inhibits breast cancer stem cell characteristics through suppression of Wnt signalling pathway by down-regulating NEK2[J]. *J. Cell. Mol. Med.* **24** (13), 7353–7369. 10.1111/jcmm.15317 (2020). 32558224 10.1111/jcmm.15317 PMC7339185

[26] Huang, Y. J. et al. Antrodia cinnamomea enhances Chemo-Sensitivity of 5-FU and suppresses colon tumorigenesis and cancer stemness via Up-Regulation of tumor suppressor miR-142-3p[J]. *Biomolecules* **9** (8). 10.3390/biom9080306 (2019). 10.3390/biom9080306 PMC6723279 31349708

[27] Troschel, F. M. et al. miR-142-3p attenuates breast cancer stem cell characteristics and decreases radioresistance in vitro[J]. *Tumour Biol.* **40** (8), 1392297777. 10.1177/1010428318791887 (2018). 10.1177/1010428318791887 30091683

[28] Gupta, P. B. et al. Stochastic state transitions give rise to phenotypic equilibrium in populations of cancer cells[J]. *Cell* **146** (4), 633–644. 10.1016/j.cell.2011.07.026 (2011). 21854987 10.1016/j.cell.2011.07.026

[29] Yang, L. et al. Targeting cancer stem cell pathways for cancer therapy[J]. *Signal. Transduct. Target. Ther.* **5** (1), 8doi. 10.1038/s41392-020-0110-5 (2020). 32296030 10.1038/s41392-020-0110-5 PMC7005297

[30] Katoh, M. & Katoh, M. WNT signaling and cancer stemness[J]. *Essays Biochem.* **66** (4), 319–331. 10.1042/EBC20220016 (2022). 35837811 10.1042/EBC20220016 PMC9484141

[31] Deng, S. et al. miRNA-192 and – 215 activate Wnt/beta-catenin signaling pathway in gastric cancer via APC[J]. *J. Cell. Physiol.* **235** (9), 6218–6229. 10.1002/jcp.29550 (2020). 32091625 10.1002/jcp.29550

[32] Si, X. et al. SOX2 upregulates side population cells and enhances their chemoresistant ability by transactivating ABCC1 expression contributing to intrinsic resistance to Paclitaxel in melanoma[J]. *Mol. Carcinog.* **59** (3), 257–264. 10.1002/mc.23148 (2020). 31883360 10.1002/mc.23148

[33] Li, L. et al. Protective autophagy decreases osimertinib cytotoxicity through regulation of stem cell-like properties in lung cancer[J]. *Cancer Lett.* **452**, 191–202. 10.1016/j.canlet.2019.03.027 (2019). 30910592 10.1016/j.canlet.2019.03.027

[34] Zhang, L. et al. Targeting breast cancer stem Cells[J]. *Int. J. Biol. Sci.* **19** (2), 552–570. 10.7150/ijbs.76187 (2023). 36632469 10.7150/ijbs.76187 PMC9830502

