# Supplementary figures and images for: MiR-142-3p targets the CXCL12/WNT/β-catenin pathway to regulate the stemness of breast cancer cells
Source: Sci Rep. 2025 Dec 29;16:3963. doi: 10.1038/s41598-025-34163-4 (PMC12855206; doi:10.1038/s41598-025-34163-4)

Fig 2b

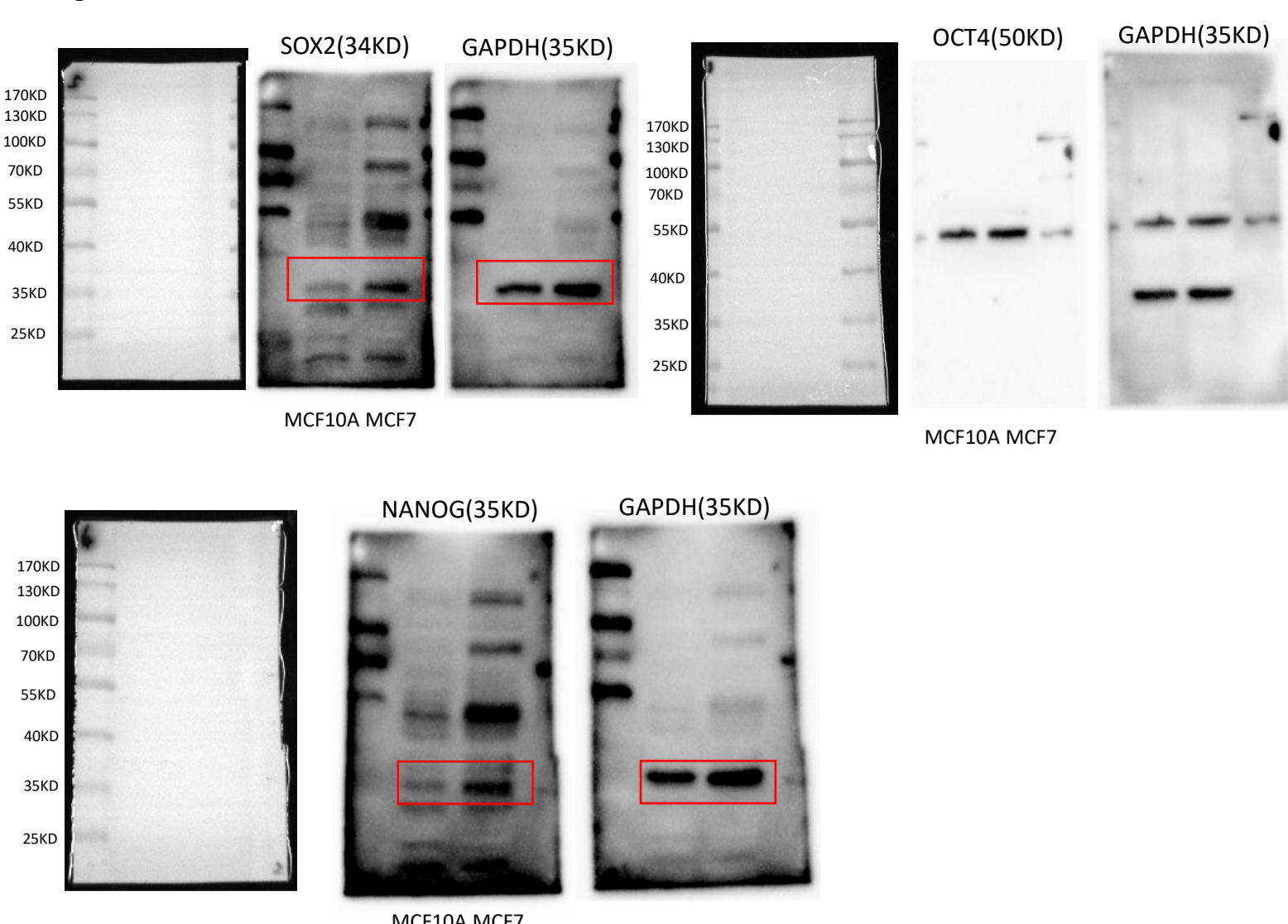

Fig 3g

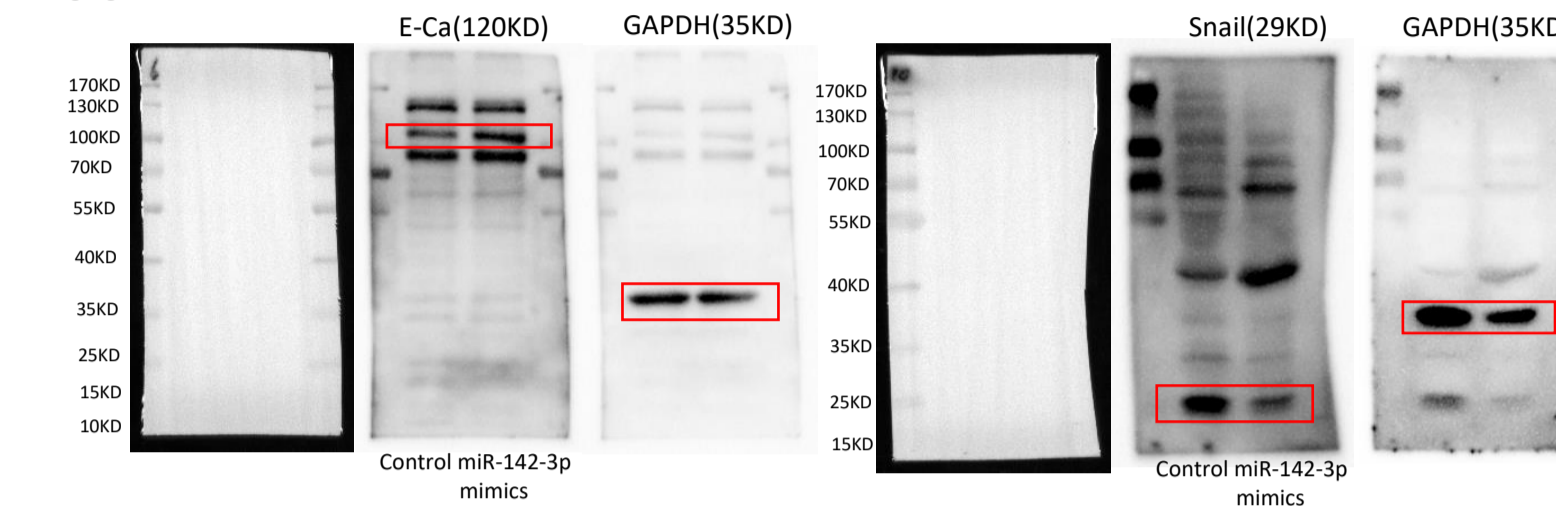

Fig 3i

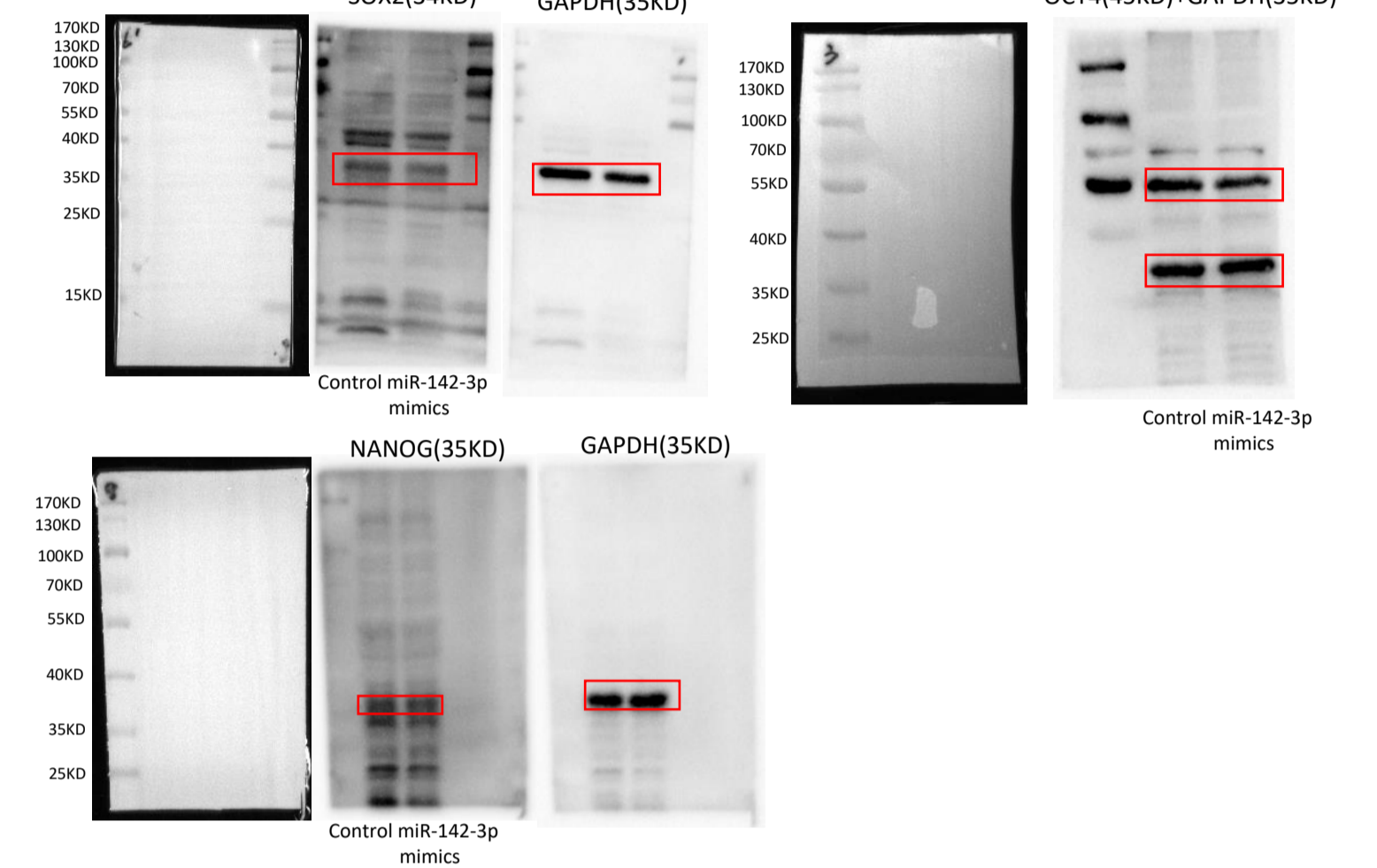

Fig 4b

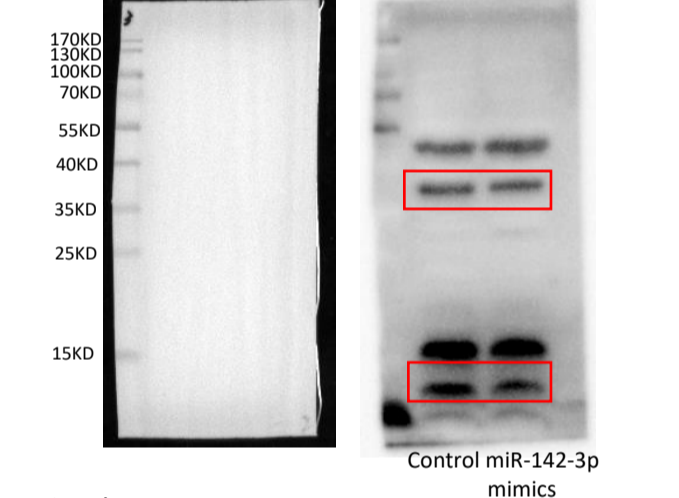

Fig 4c

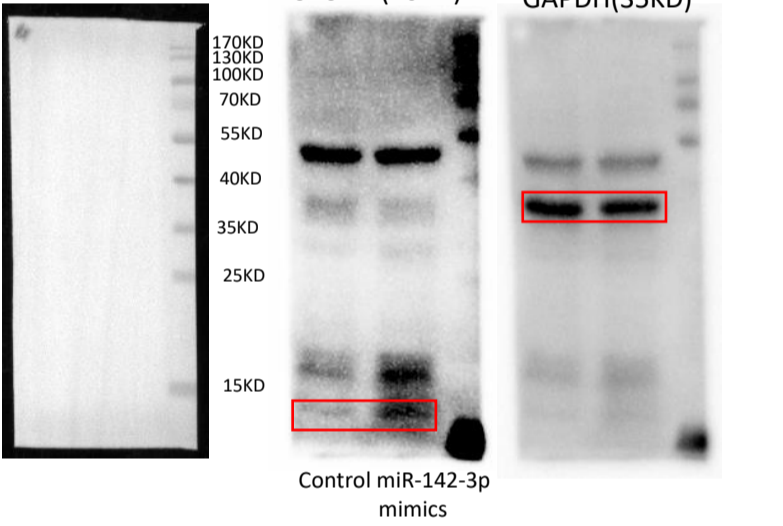

Fig 5d

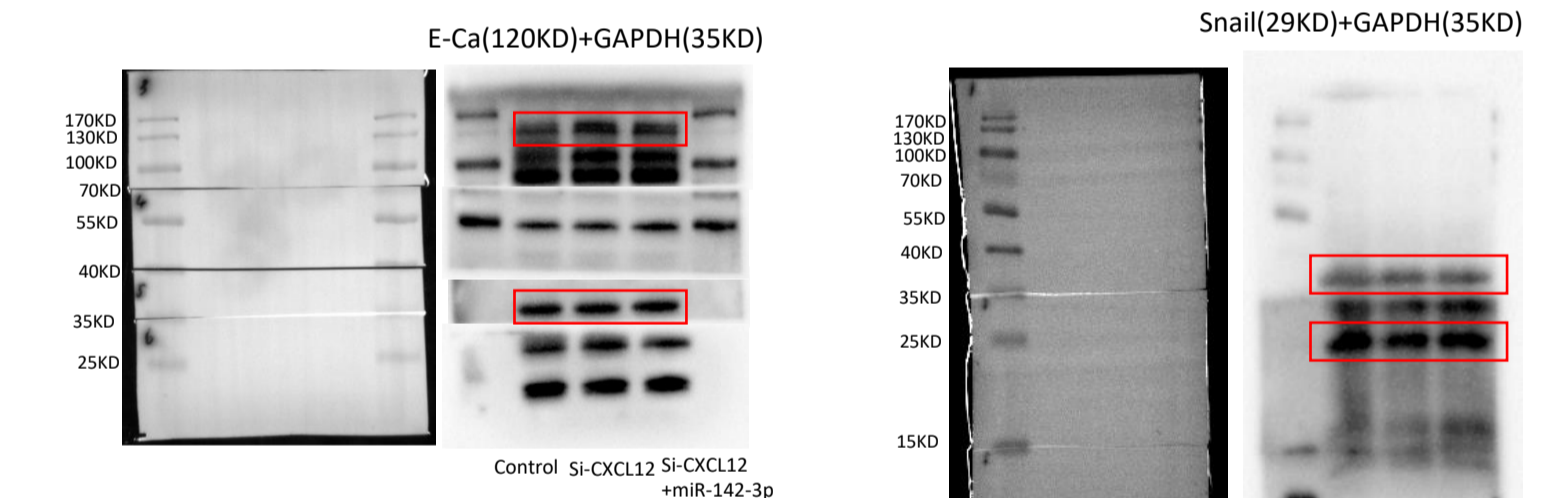

Fig 5f

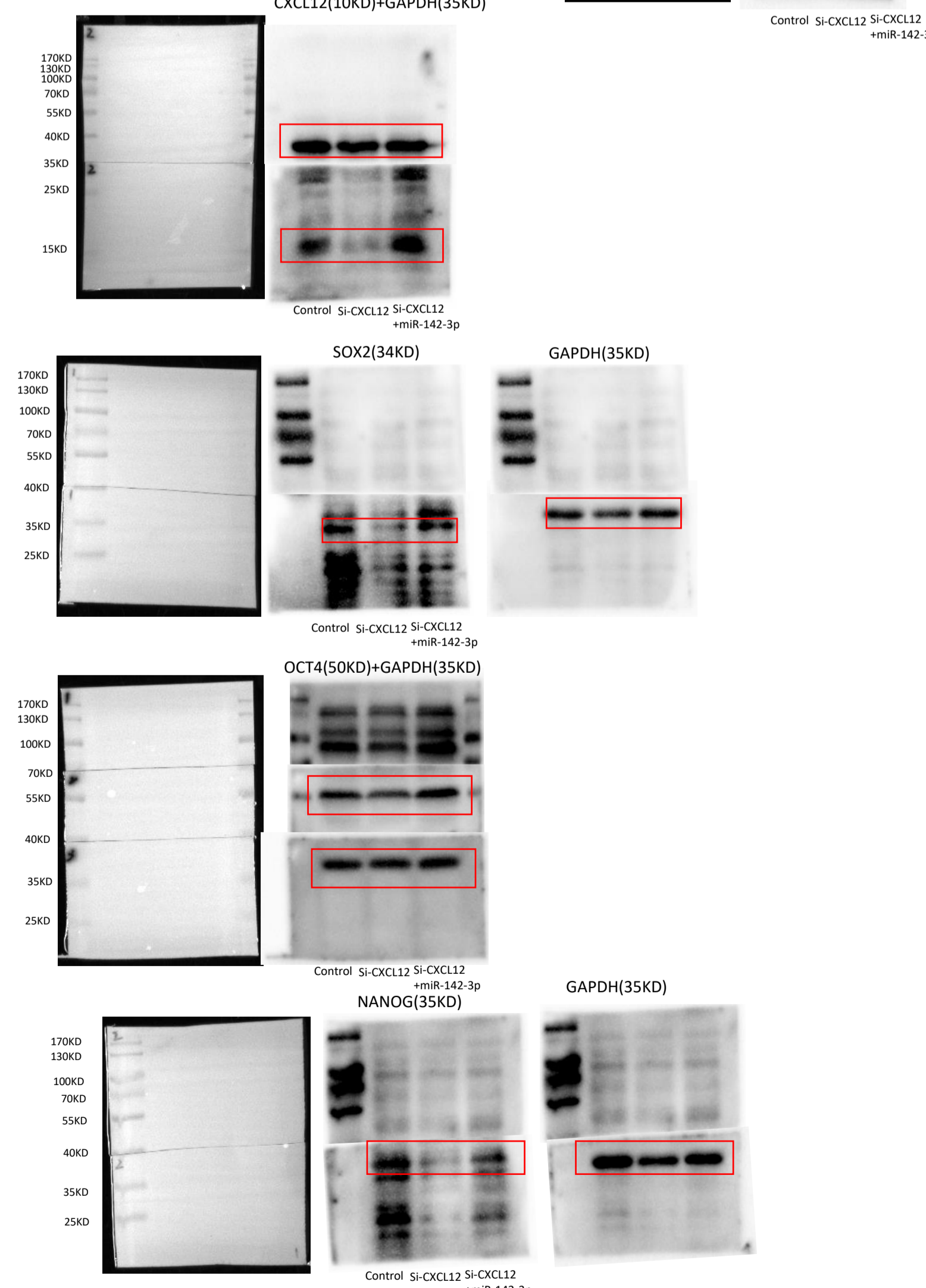

Fig 5g

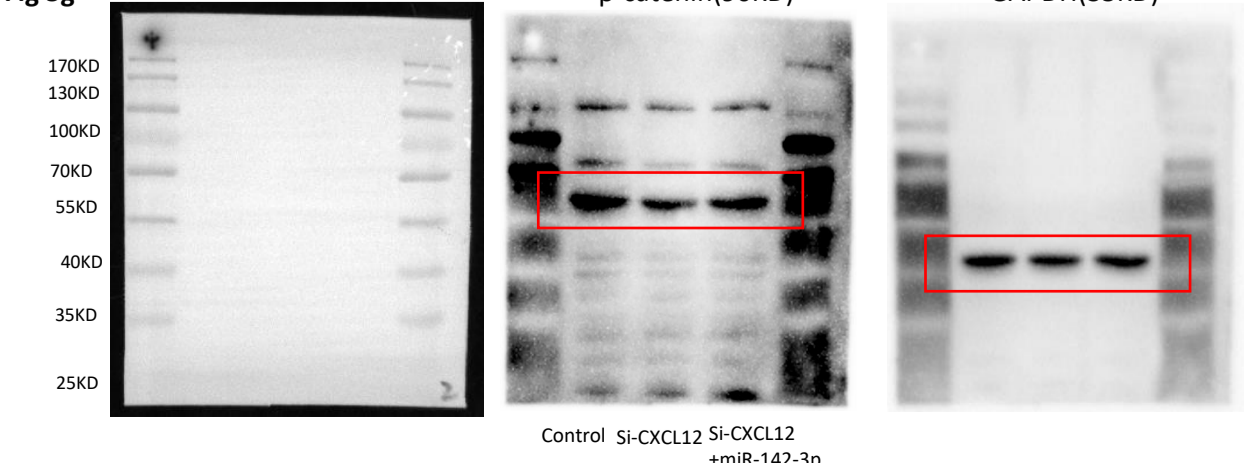

Fig 6c

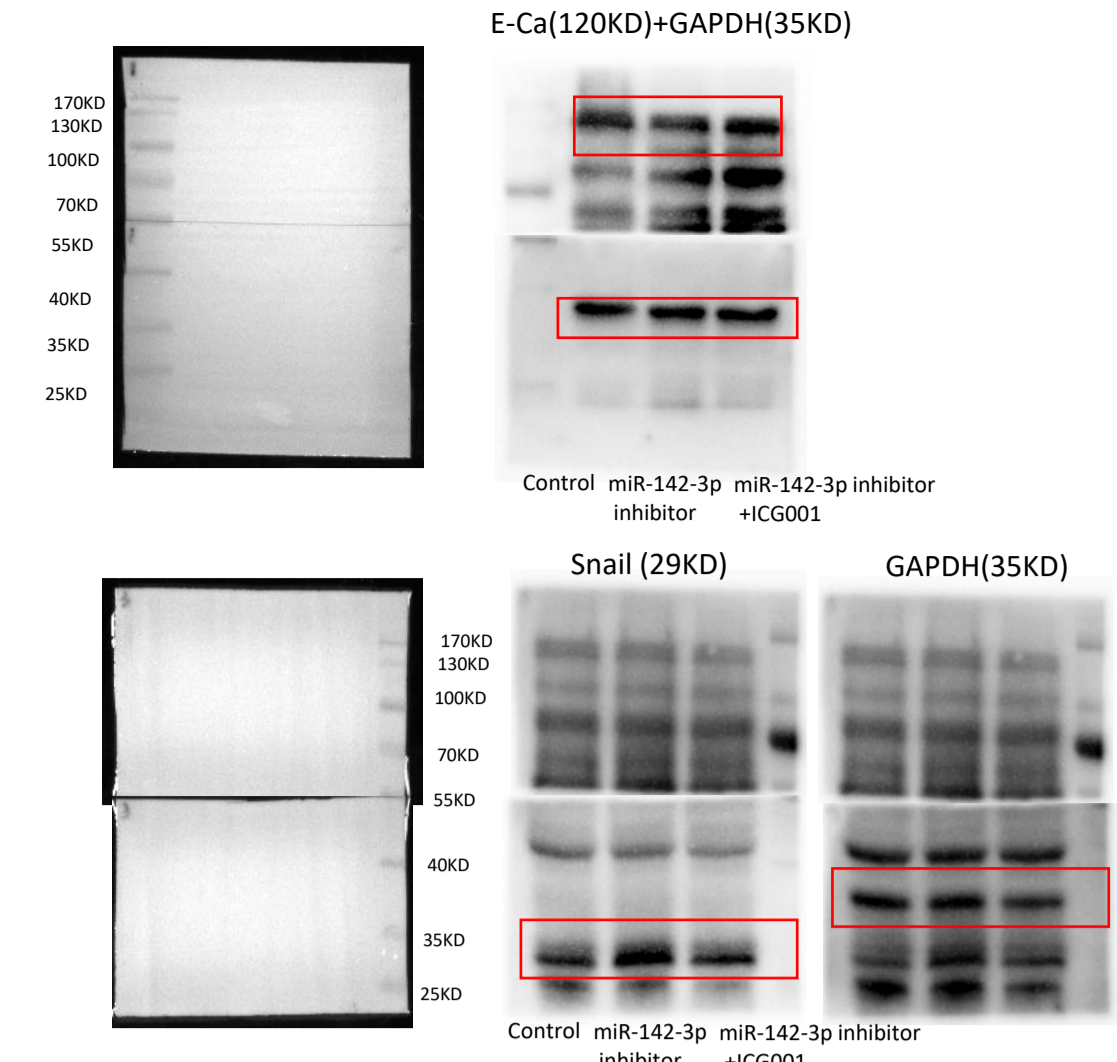

Fig 6e

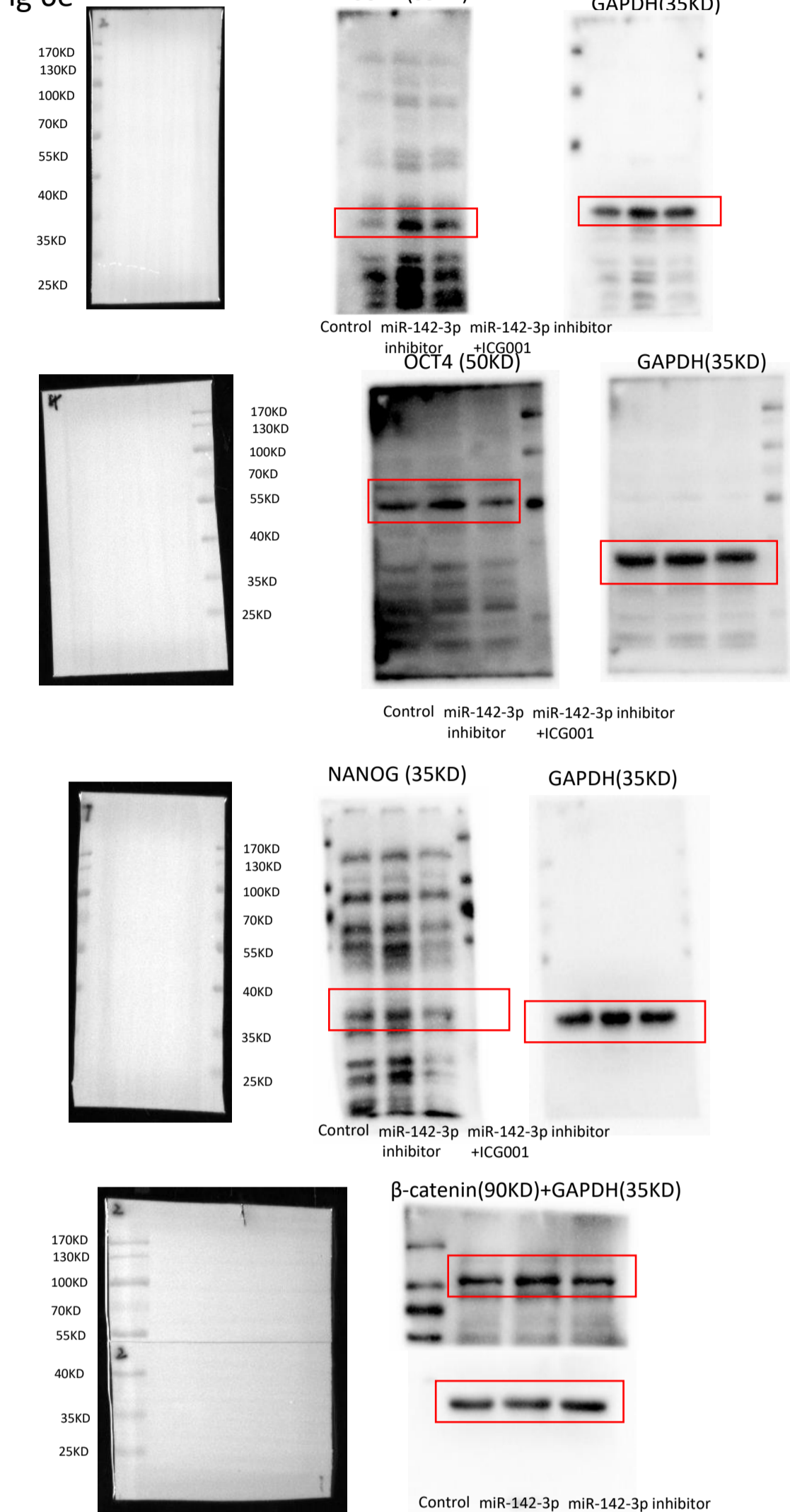

Fig 7a

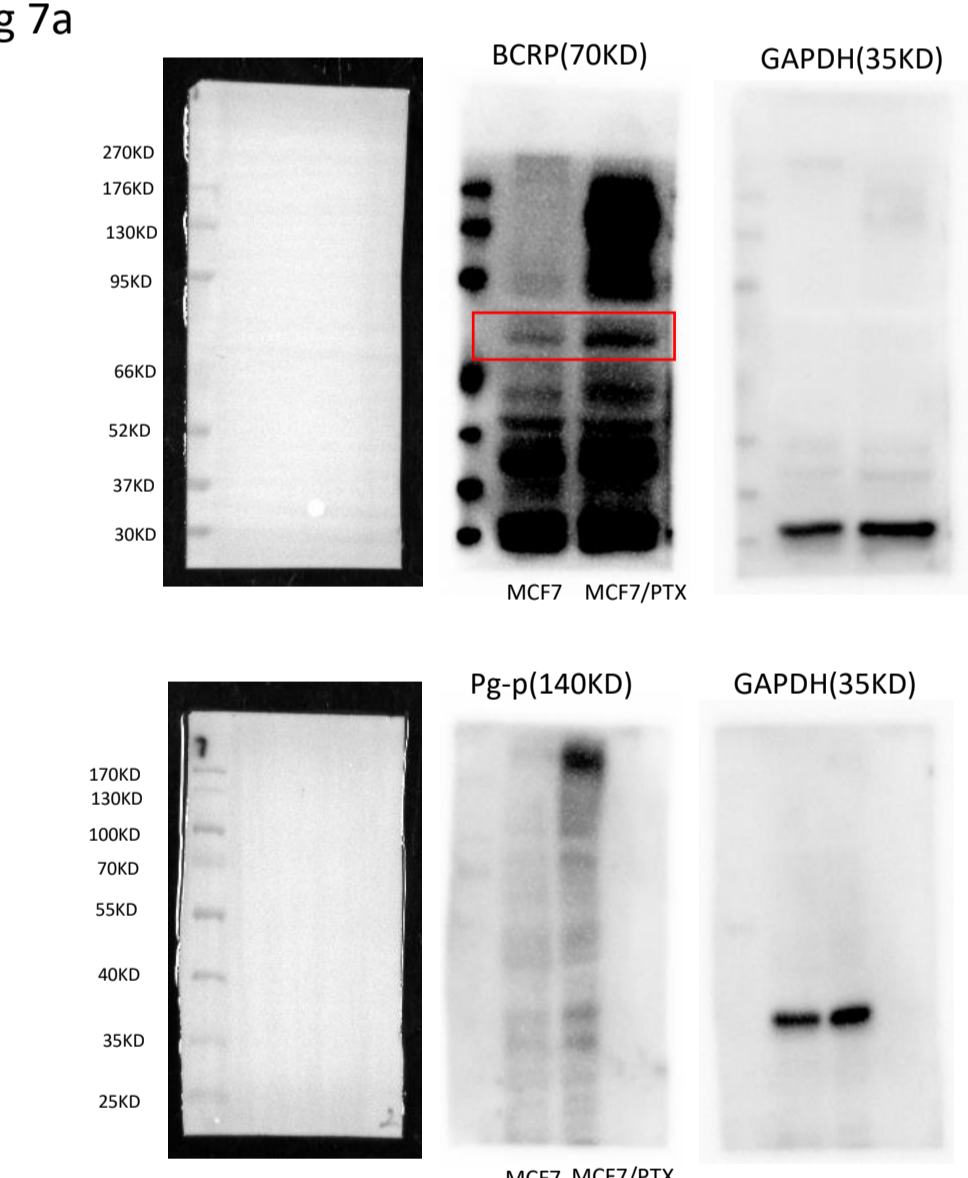

Fig 7d

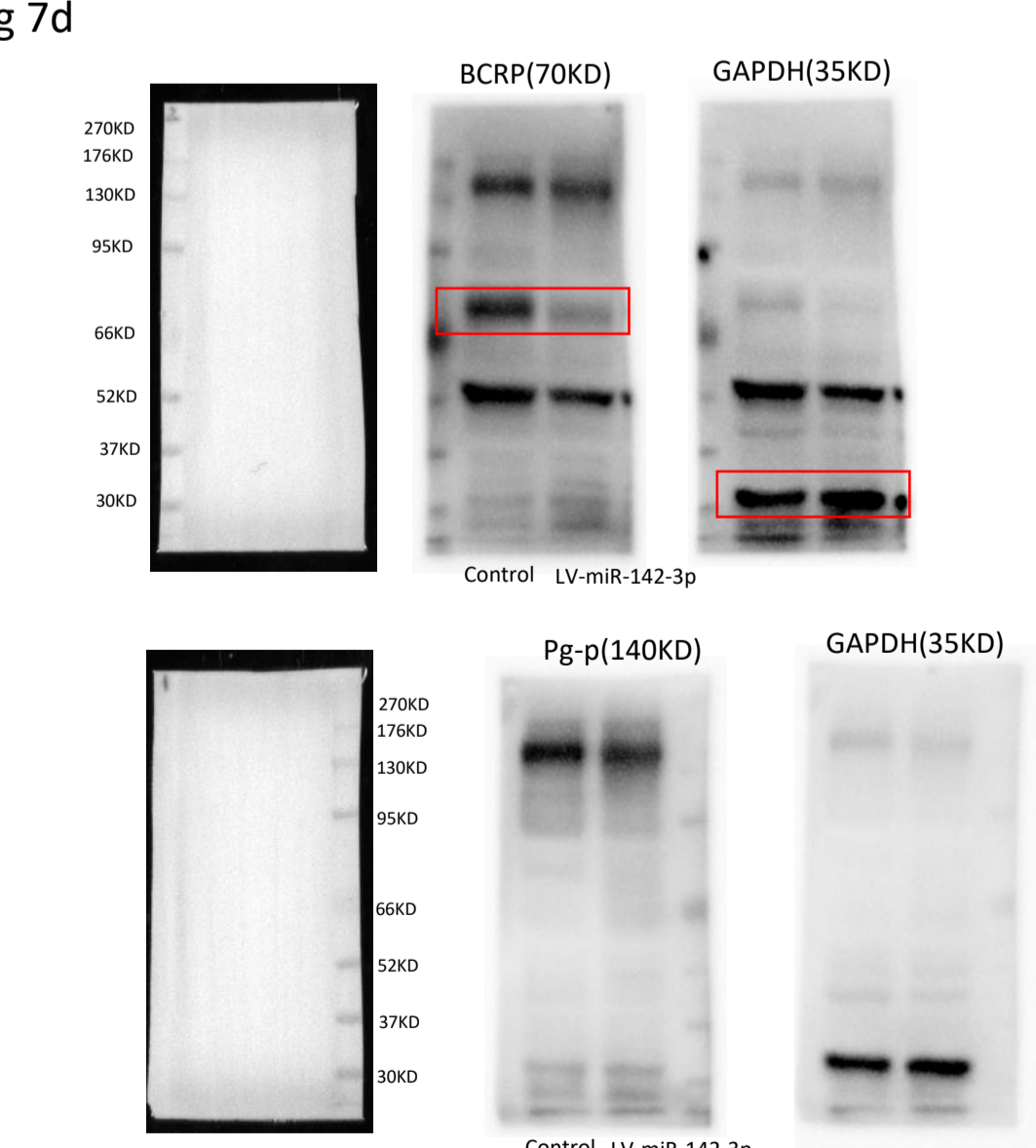

Fig 8a

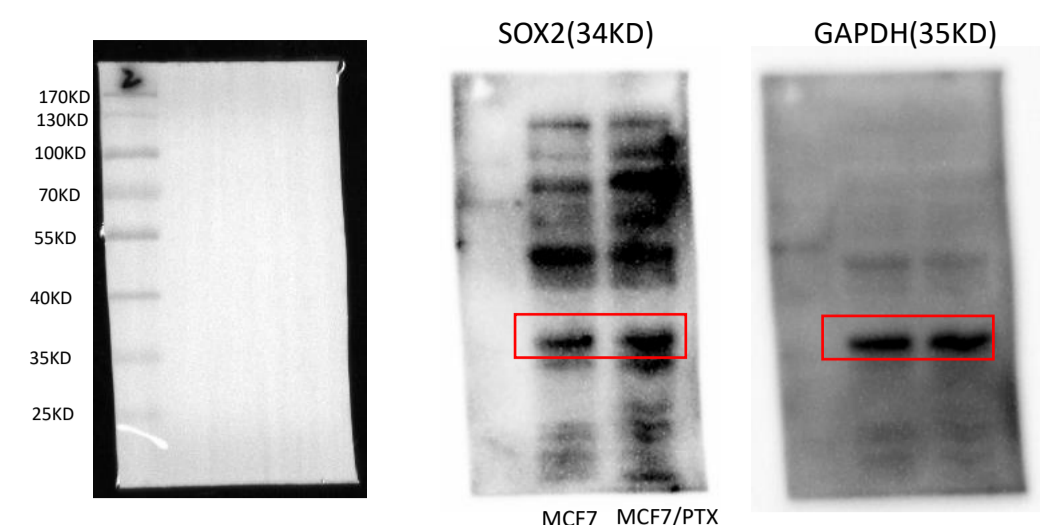

Fig 8b

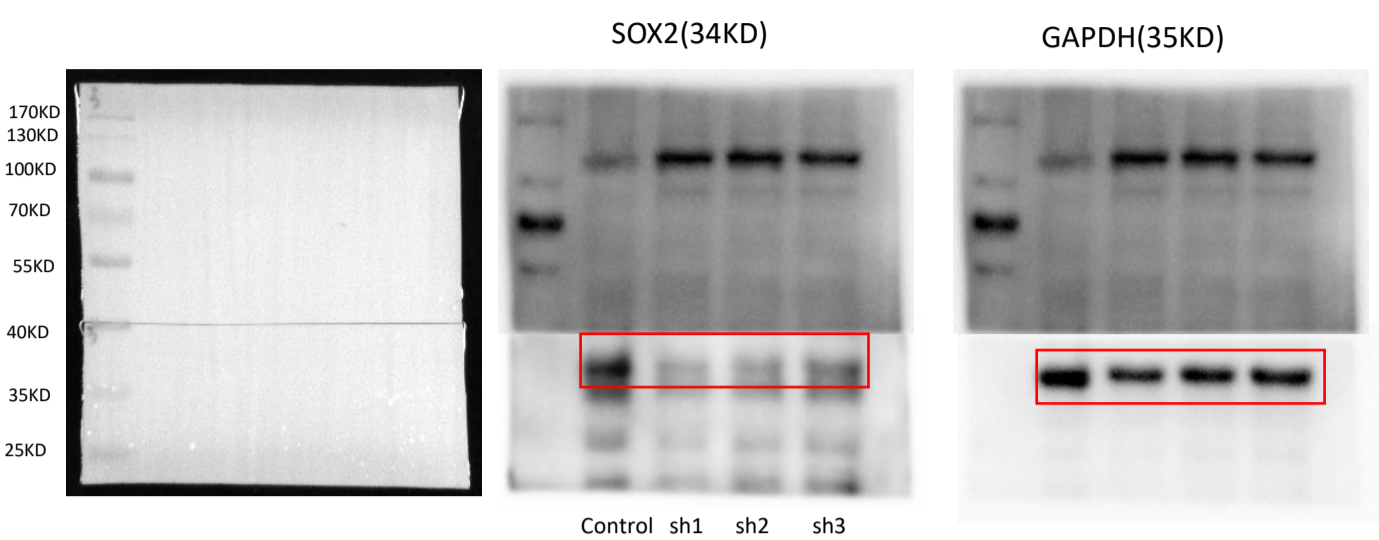

Fig 8c

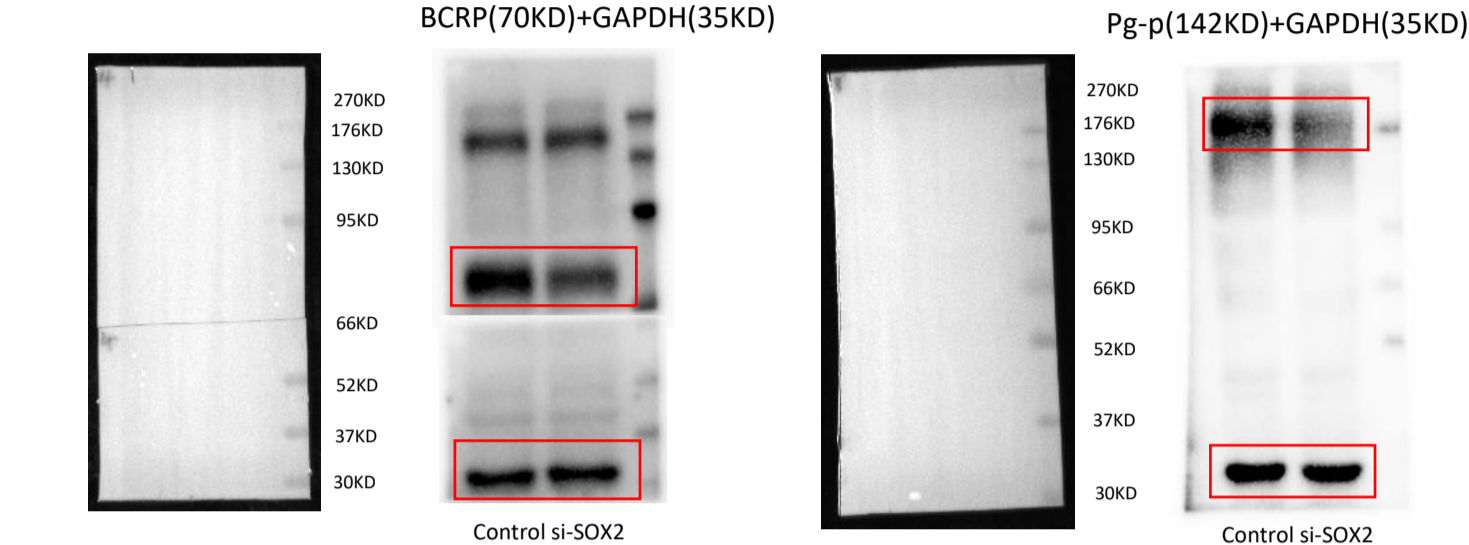

Fig 8g

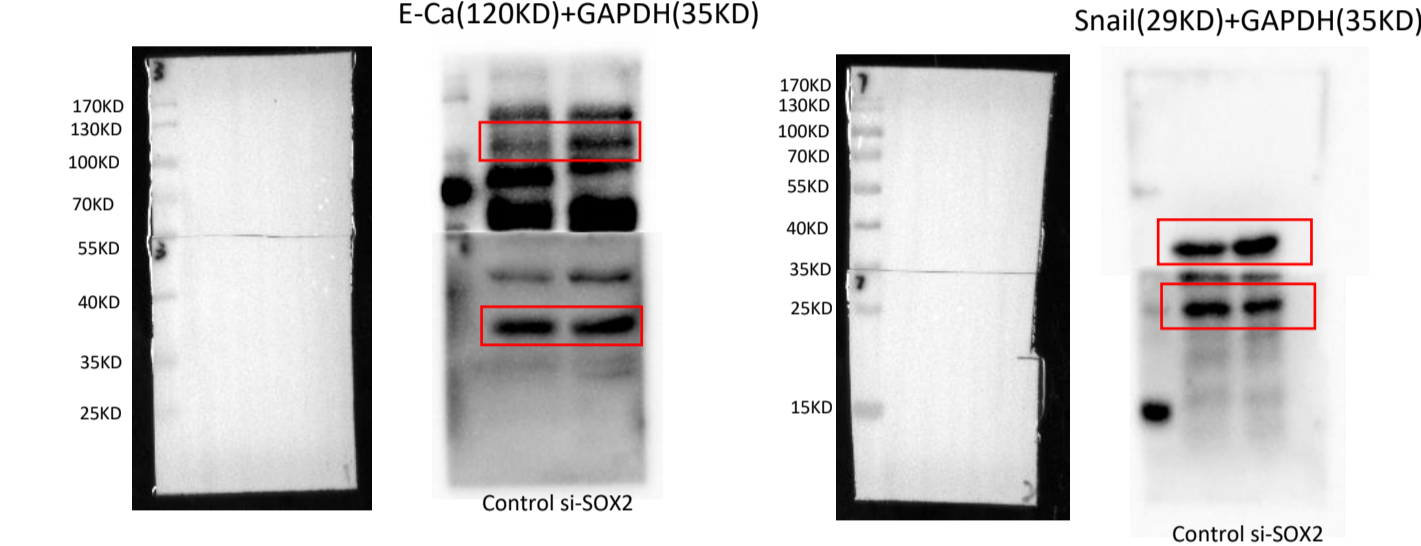

Supplement: Supplementary file 1 — Supplementary Material 1 [file 41598_2025_34163_MOESM1_ESM.pdf]
